# ABC triblock bottlebrush copolymer-based injectable hydrogels: design, synthesis, and application to expanding the therapeutic index of cancer immunochemotherapy†

**DOI:** 10.1039/d0sc02611e

**Published:** 2020-06-01

**Authors:** Farrukh Vohidov, Lauren E. Milling, Qixian Chen, Wenxu Zhang, Sachin Bhagchandani, Hung V.-T. Nguyen, Darrell J. Irvine, Jeremiah A. Johnson

**Affiliations:** Department of Chemistry, Massachusetts Institute of Technology Massachusetts 02139 USA jaj2109@mit.edu; Koch Institute for Integrative Cancer Research, Massachusetts Institute of Technology Cambridge Massachusetts 02139 USA djirvine@mit.edu

## Abstract

Bottlebrush copolymers are a versatile class of macromolecular architectures with broad applications in the fields of drug delivery, self-assembly, and polymer networks. Here, the modular nature of graft-through ring-opening metathesis polymerization (ROMP) is exploited to synthesize “ABC” triblock bottlebrush copolymers (TBCs) from polylactic acid (PLA), polyethylene glycol (PEG), and poly(*N*-isopropylacrylamide) (PNIPAM) macromonomers. Due to the hydrophobicity of their PLA domains, these TBCs self-assemble in aqueous media at room temperature to yield uniform ∼100 nm micelles that can encapsulate a wide range of therapeutic agents. Heating these micellar solutions above the lower critical solution temperature (LCST) of PNIPAM (∼32 °C) induces the rapid formation of multi-compartment hydrogels with PLA and PNIPAM domains acting as physical crosslinks. Following the synthesis and characterization of these materials *in vitro*, TBC micelles loaded with various biologically active small molecules were investigated as injectable hydrogels for sustained drug release *in vivo*. Specifically, intratumoral administration of TBCs containing paclitaxel and resiquimod—the latter a potent Toll-like receptor (TLR) 7/8 agonist—into mice bearing subcutaneous CT26 tumors resulted in a significantly enhanced therapeutic index compared to the administration of these two drugs alone. This effect is attributed to the TBC hydrogel maintaining a high local drug concentration, thus reducing systemic immune activation and local inflammation. Collectively, this work represents, to our knowledge, the first example of thermally-responsive TBCs designed for multi-compartment hydrogel formation, establishing these materials as versatile scaffolds for self-assembly and drug delivery.

## Abstract 2

Triblock bottlebrush copolymers with an "ABC" sidechain sequence form thermally reversible hydrogels for sustained delivery of paclitaxel and resiquimod, improving the efficacy and safety of this potent cancer immunochemotherapy.

## Graphical abstract

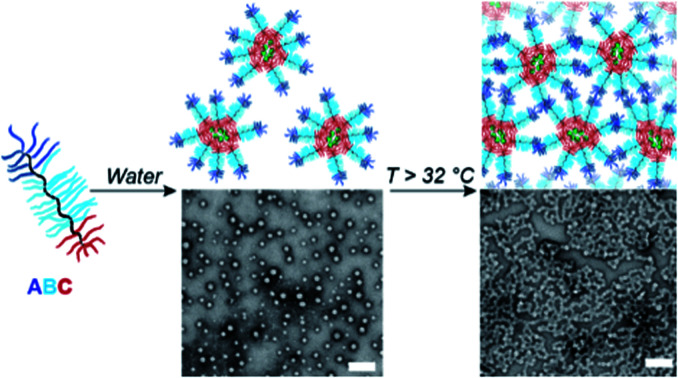

## Introduction

Driven by advances in their synthesis, bottlebrush polymers have experienced a renaissance in recent years, with applications ranging from drug delivery and molecular imaging to self-assembly and polymer network fabrication.^1–6^ Of the strategies for bottlebrush polymer synthesis,^7–10^ graft-through polymerization of macromonomers (MMs) offers advantages of complete backbone functionalization and facile access to multi-block copolymers.^11–13^ Ring-opening metathesis polymerization (ROMP) of norbornene-based MMs is perhaps the most useful method for graft-through bottlebrush polymer synthesis thanks to its efficiency and tolerance toward a wide range of functional groups; this approach has been used to generate bottlebrush-based polymer architectures with unique self-assembly, imaging, and drug delivery capabilities.^14–17^ Despite these advances, and given the well-established utility of triblock copolymers in self-assembly and biomedicine,^18–21^ there are limited examples of triblock bottlebrush copolymers (TBCs).^22–24^ For instance, Bates and coworkers prepared two-component “ABA” TBCs from polystyrene and polyethylene glycol (PEG) MMs and demonstrated their utility as Li-ion conductors.^22^ Additionally, reports on the solution^12,23,25–28^ and bulk^29–31^ self-assembly of ABA, as well as three-component “ABC” TBCs, have hinted at exciting opportunities for this class of macromolecular architectures.

**Fig. 1:**
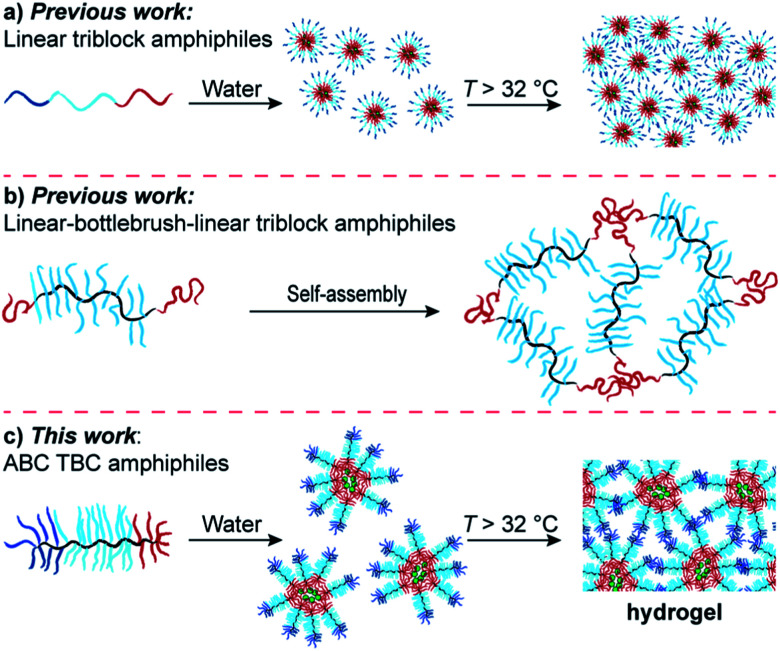
(a) Linear triblock copolymer amphiphiles with thermoresponsive character. (b) Linear–bottlebrush–linear triblock copolymer networks (c) “ABC” triblock bottlebrush copolymer (TBC) amphiphiles and thermally responsive hydrogels described in this work.

We reasoned that appropriately designed ABC TBCs could serve as macromolecular precursors to injectable hydrogels with advantages including ease-of-synthesis, low critical micelle concentrations (CMCs),^32^ and precise control over their compartment domain sizes. We were inspired by the pioneering work of Tirrell and coworkers, who showed that physical hydrogels composed of linear ABC triblock protein macromers, where A and C are dissimilar, non-associating protein domains and B is a linker domain, eroded significantly more slowly than ABA or CBC hydrogels.^33^ This result was rationalized based on the unique topology of the ABC network,^34,35^ which cannot form mechanically defective primary loops.^36,37^ Similarly, Lodge and coworkers reported linear ABC triblock copolymers with hydrophobic A, hydrophilic B, and thermoresponsive C blocks that formed micelles in water at room temperature and underwent physical gelation upon heating above the lower-critical solution temperature (LCST) of the C block (Fig. 1a). Here again, materials derived from ABC triblock copolymers were more mechanically robust than analogous ABA and CBC gels, presumably due to a reduction of primary loops.^38,39^ Duvall and coworkers demonstrated the utility of linear ABC triblock copolymers with tunable degradation properties for controlled drug delivery applications.^40,41^ Recently, Sheiko and coworkers reported on the development of linear–bottlebrush–linear “ABA” triblock copolymer elastomers where the central bottlebrush domain imbued the materials with strain stiffening behaviors analogous to biological tissues (Fig. 1b).^42^ This study highlights the broad tunability of bottlebrush polymer networks, as variables such as grafting density, backbone length, and sidechain length all can be modified to achieve predictable material properties. Nevertheless, bottlebrush polymer materials based on the unique ABC topology (Fig. 1c) have not, to our knowledge, been reported in the context of supramolecular hydrogels, nor have they been examined in biomedical applications such as drug delivery. We envisioned that due to their unique ability to form multi-compartment networks, such materials could be especially useful as injectable hydrogels for local drug delivery. Unlike their ABA or AB counterparts, ABC copolymers enable separate micellization and gelation steps for straightforward drug encapsulation followed by formation of local drug depots at the site of injection, respectively. In the context of cancer immunotherapy^43–46^ where, for example, controlled release of agents that can generate cancer cell antigens and activate dendritic cells (DCs) selectively within the tumor microenvironment, ABC TBCs could significantly improve both the efficacy and safety of immunotherapies.^47,48^

**Fig. 2:**
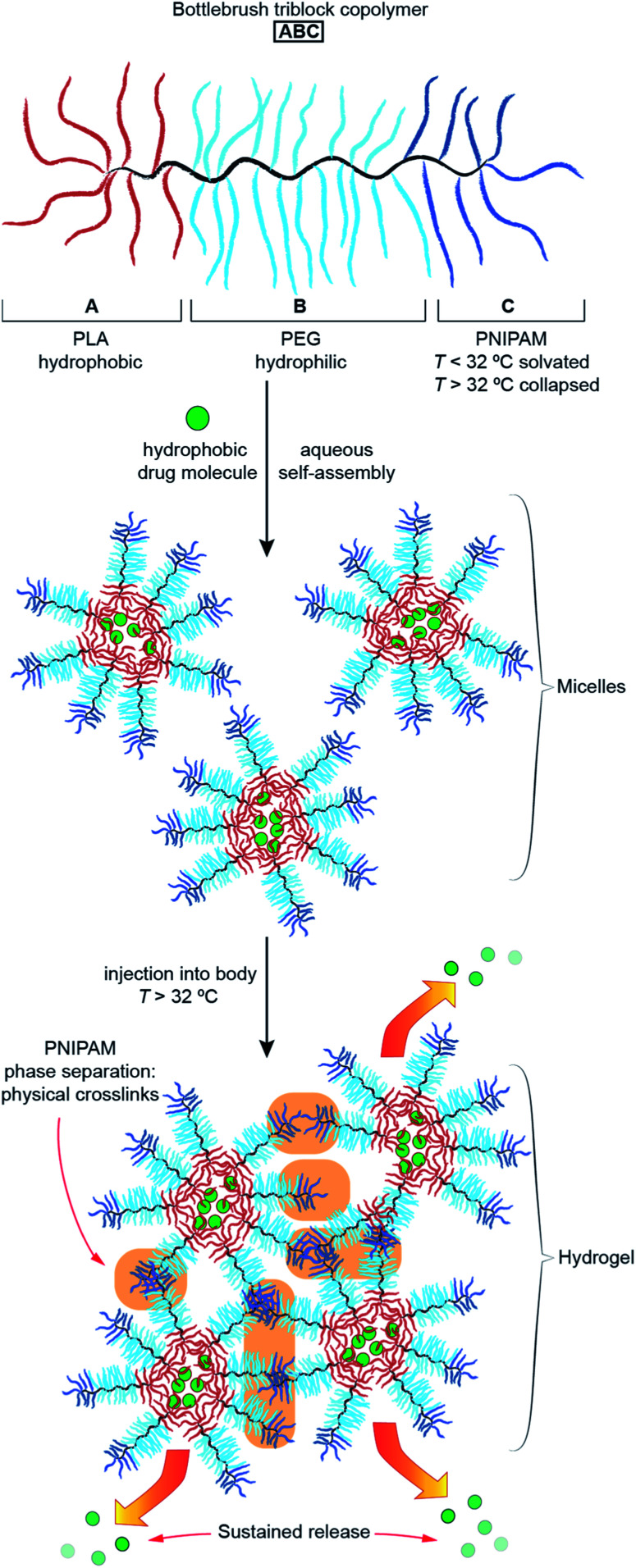
Thermoresponsive ABC TBCs for local drug delivery. At room temperature, the TBCs self-assemble into micelles encapsulating hydrophobic drug molecules. Upon *in vivo* administration, PNIPAM phase separation induces hydrogel formation. Sustained, local release is achieved by diffusion of drug molecules from the hydrogel.

**Fig. 3:**
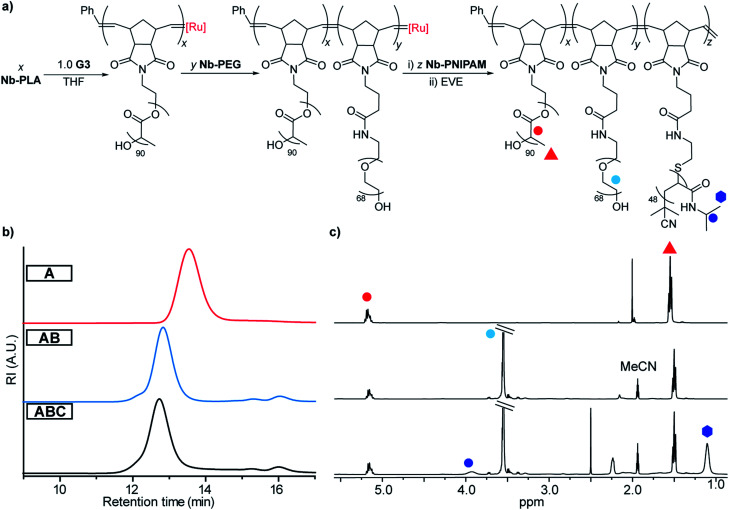
(a) Sequential ROMP used for synthesis of TBCs. (b) Size-exclusion chromatography (SEC) analysis of sequential TBC growth. (c) Nuclear magnetic resonance (^1^H NMR) of PLA bottlebrush polymer (**A**), PLA-*b*-PEG bottlebrush polymer (**AB**), and PLA-*b*-PEG-*b*-PNIPAM TBC (**ABC**).

With this goal in mind, we describe herein the design and synthesis of ABC TBCs *via* sequential ROMP of polylactic acid (PLA), polyethylene glycol (PEG), and poly(*N*-isopropylacrylamide) (PNIPAM) MMs (Fig. 2 and 3). Driven by the hydrophobicity of their PLA domains, TBCs with an ABC block sequence are shown to self-assemble in aqueous solutions at room temperature, forming uniform ∼100 nm nanoparticles that can encapsulate and release small-molecule drugs (Fig. S1†) including paclitaxel (PTX), SN-38, resiquimod (R848), and gemcitabine, as well as a model protein (ovalbumin). Moreover, when heated above the LCST of PNIPAM (∼32 °C), which is near physiological temperature (∼37 °C), these TBC micelles rapidly formed hydrogels comprising a physically crosslinked network of hydrophobic PLA and PNIPAM domains connected by PEG domains (Fig. 1c and 2). TBC hydrogels loaded with PTX and R848 were administered intratumorally to immunocompetent mice bearing subcutaneous CT26 hind-flank tumors. The therapeutic index of the TBC hydrogel-based drug combination was improved compared to the combination of free PTX and R848, as determined by tumor volume measurements, quantification of systemic cytokines, and histology. In addition, cured mice re-challenged with CT26 cells did not develop new tumors, suggesting that the TBC hydrogel-mediated delivery facilitates an adaptive immune response. Altogether, this work establishes ABC TBCs as modular scaffolds for the formation of hydrogels with a range of potential controlled release applications.

## Design and synthesis of thermoresponsive TBC amphiphiles

To access the desired TBCs, we first prepared *exo*-norbornene imide-terminated MMs based on PLA (**Nb-PLA**), PEG (**Nb-PEG**), and PNIPAM (**Nb-PNIPAM**). **Nb-PLA** and **Nb-PEG** were synthesized following previously reported methods,^49,50^ whereas **Nb-PNIPAM** was synthesized by coupling of commercially available 5.5 kDa amine-terminated PNIPAM to a previously reported norbornene-*N*-hydroxysuccinimidyl^50^ ester (see ESI† for details). ROMP of these MMs was initiated by Grubbs 3^rd^ generation bispyridyl complex (**G3**).

First, a TBC sample referred to as “**ABC**” was prepared through sequential copolymerization of **Nb-PLA**, **Nb-PEG**, and **Nb-PNIPAM** (Fig. 3a and Table 1). Size exclusion chromatography (SEC) traces obtained after consumption of **Nb-PLA** (red), **NB-PEG** (blue) and **Nb-PNIPAM** (black) confirmed the incorporation of each block into **ABC** (Fig. 3b). Following quenching with excess ethyl vinyl ether (EVE), the reaction mixture was dialyzed against milliQ water (50 kDa molecular weight cutoff (MWCO) dialysis tubing), concentrated (using a 100 kDa MWCO centrifugal filter), and lyophilized to dryness. SEC analysis of the isolated **ABC** revealed a unimodal peak with no residual macromonomer (Fig. S2†). The number-average molar mass (*M*_n_) of **ABC** was estimated by assuming complete conversion of **Nb-PLA** (as observed by SEC) and comparing the ^1^H NMR resonances for the PEG and PNIPAM segments to those of PLA (Fig. 3c and Table 1); close agreement between the MM feed ratios (*e.g.*, theoretical *M*_n_) and experimentally measured block content was observed. To study the impact of block size and sequence on the properties of TBCs, a series of related copolymers was synthesized for comparison to **ABC**: “**CBA**” was prepared *via* sequential ROMP of the same MMs in the same ratios as **ABC** but in the reverse order; “**ABC-stat**” was synthesized by mixing the three MMs with **G3** providing a statistical copolymer with the same overall composition as **ABC**; lastly, a diblock bottlebrush copolymer “**AB**” lacking a PNIPAM block was synthesized as a non-thermoresponsive analogue of **ABC**. SEC, ^1^H NMR, differential scanning calorimetry (DSC), and thermogravimetric analysis (TGA) supported the proposed structures of these polymers (Table 1 and Fig. S3–S9†).

**Table 1:** Compositions of bottlebrush copolymers

Entry	Copolymer	*N*_(PLA 6.6 k)_ a	*N*_(PEO 3 kDa)_ b	*N*_(PNIPAM 5.5 k)_ b	*M*_n_ (kg mol^−1^)^b^	*Đ*
**AB**	PLA-*b*-PEG	7.6	43.2	—	179	1.12
**ABC**	PLA-*b*-PEG-*b*-PNIPAM	7.6	43.8	8.6	229	1.25
**CBA**	PNIPAM-*b*-PEG-*b*-PLA	7.6^a^	40^a^	10^a^	225	1.35
**ABC-stat**	PLA-*co*-PEG-*co*-PNIPAM	7.6	41.4	8.2	219	1.21

a Assumed quantitative incorporation based on SEC analysis.

b Based on ^1^H NMR peak integrations relative to PLA content.

## Aqueous self-assembly and thermoresponsive behavior of TBCs

**Fig. 4:**
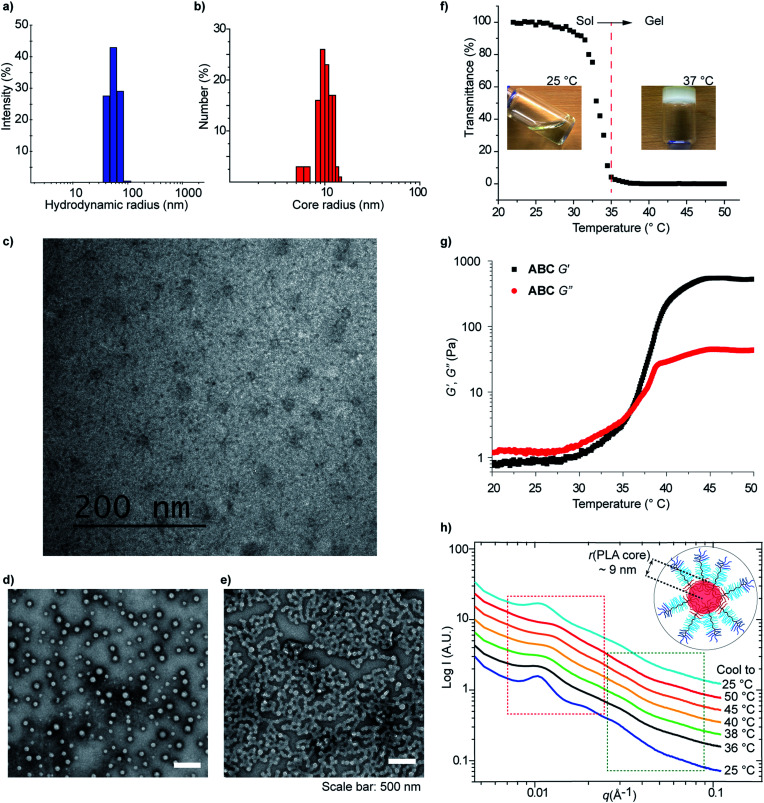
(a) DLS analysis of aqueous self-assembly of **ABC** triblock copolymer. (b) Core size distribution of micelles of **ABC** TBC measured from cryoTEM images. (c) **ABC** micellar solution imaged in native state by cryoTEM. (d and e) TEM images of dilute **ABC** micelle solutions prepared with UO_2_(OAc)_2_ stain at 25 °C and 37 °C. (f) Temperature-dependent light transmission (640 nm) of **ABC** micellar solution (10 wt%). (g) Temperature sweep rheology of **ABC** micellar solution (10 wt%). (h) Variable-temperature small-angle X-ray scattering (SAXS) curves of **ABC** micellar solution (10 wt%).

The solution self-assembly of these TBCs (1 mg mL^−1^ in water) was studied using dynamic light scattering (DLS) and transmission electron microscopy (TEM) (Fig. 4 and S10–S12†). Samples of TBC micelles were prepared by slow dilution of rapidly stirring DMSO solutions with water or saline buffer (to ∼1% DMSO by volume). Diblock **AB**, in contrast, was dissolved directly in water at 50 °C and no LCST behavior was observed. For these samples, DLS analysis revealed the presence of micelles with hydrodynamic diameters (*D*_h_) ranging from 90–119 nm (Table 2, Fig. 4a and S10†). As expected, **ABC** and **CBA** generated micelles of comparable sizes (113 nm, PDI: 0.05 and 120 nm, PDI: 0.07, respectively), consistent with their similar compositions. In addition, **AB** formed smaller micelles (93 nm, 0.08 PDI) as would be expected given its lack of a PNIPAM block. In contrast, **ABC-stat** formed considerably smaller (*D*_h_ = 45 nm, PDI: 0.17) assemblies compared to its TBC counterparts, highlighting the importance of sequence in programming TBC self-assembly. Variable-temperature DLS experiments revealed that dilute **ABC** micelles undergo reversible aggregation upon heating above the LCST of PNIPAM (Fig. S11†), presumably due to the formation of micelleplexes driven by PNIPAM hydrophobic interactions above its LCST.

**Table 2:** Aqueous self-assembly and drug encapsulation efficiency of bottlebrush copolymers

Micelle	*D*_h_ (nm)	PDI	Encapsulation efficiency^a^ (%)
**AB**	93	0.08	—
**ABC**	113	0.05	—
**CBA**	120	0.07	—
**ABC-stat**	45	0.17	—
Dexamethasone/**ABC**	92	0.1	78
Ibuprofen/**ABC**	108	0.09	65
Ofloxacin/**ABC**	113	0.16	63
Paclitaxel (PTX)/**ABC**	104	0.14	91
Resiquimod (R848)/**ABC**	118	0.15	71

a Drug encapsulation efficiency based on % of drug (5 mg) retained in **ABC** TBC (50 mg).

Cryogenic transmission electron microscopy (cryoTEM) imaging confirmed the assembly of **ABC** into micelles with hydrophilic blocks forming a shell around well-resolved cores (Fig. 4b, c and S12†). The average micelle core size, as determined from the cryoTEM images, was 20 ± 3 nm (Fig. 4b). Assuming tight packing of the PLA blocks in the micelle cores and using the density of PLA (1.24 g cm^−3^), we estimate an average micelle is formed from approximately 100 TBC macromolecules.

Additionally, micelles derived from **ABC** were imaged by conventional TEM following drop-casting onto a carbon film-coated copper grid and negative staining (uranyl acetate), revealing particle sizes (102 ± 19 nm) that closely matched the *D*_h_ values determined by DLS (Fig. 4d). When analogous samples were dried at an elevated temperature (>37 °C) on the TEM grid, the formation of higher-order assemblies *via* fusion of micelles was directly observed (Fig. 4e).

Next, we investigated the thermally-induced gelation of aqueous solutions of a higher concentration (10 wt%) of **ABC**. At room temperature, the 10 wt% **ABC** solution is a clear liquid; upon heating to >32 °C this solution converts into an opaque, free-standing hydrogel (Fig. 4f). Temperature sweep (1 °C min^−1^) shear rheology demonstrated that at room temperature the 10 wt% **ABC** solution behaves like a viscous liquid with a greater loss modulus (*G*′′) than storage modulus (*G*′) (Fig. 4g). Both *G*′ and *G*′′ increased with increasing temperature, however, and *G*′ increased much faster; the two values intersect at 36 °C, indicative of thermally induced gelation. At temperatures greater than 37 °C, the material behaves like a soft elastic solid (*G*′ > *G*′′).

Characterization of **ABC** solutions using small angle X-ray scattering (SAXS) painted a similar picture. At room temperature, the scattering profile of an aqueous solution of **ABC** (10 wt%) displayed a group of broad peaks at low *q* values (*q* < 0.02 Å^−1^, red box in Fig. 4h) that are assigned to the structural factors, which are related to the inter-particle spatial relationship. The inter-domain spacing (*d*) calculated using *d* = 2π/*q* was found to be ∼60 nm, which suggests that the hydrophilic shells of these **ABC** micelles (*D*_h_ = 110 nm) overlap at this concentration (10 wt%). As expected, *d* correlated inversely with **ABC** micelle concentration (Fig. S13†). The small peaks in the higher *q* region (green box in Fig. 4d) are attributed to the form factor of the PLA core. By fitting with a hard sphere model, the average core radius was estimated to be ∼9 nm, which agrees well with the cryoTEM results (Fig. 4b). Temperature-dependent SAXS revealed clear changes in the scattering curves, most notably a shift of the structure factor peaks to higher *q* (smaller *d*), which is likely a consequence of the collapse of the PNIPAM phase and inter-micelle crosslinking. Upon cooling the sample, the SAXS profile returned to nearly the same form as in the initial state, indicating that the shape and size of the micelles are maintained following heating and cooling and that the gelation process is reversible.

Lastly, thermal gelation of **ABC** was investigated *in vivo*. A solution (10 wt% in saline buffer) of fluorescently labeled (cyanine 7.5) TBC sample “**ABC-Cy7.5**” was injected subcutaneously into the hind flank of healthy balb/c mice (*n* = 3) with their hair shaved around the injection site. The mice were imaged (IVIS; *λ*_ex/em_ = 745/800 nm) shortly after injection and again after 24 h. A fluorescently labeled diblock bottlebrush “**AB-Cy7.5**” lacking a PNIPAM domain was used as a control sample. Fig. S14† shows that **ABC-Cy7.5** formed a localized patch at the injection site with no signs of spreading after 24 h. In contrast, after 24 h **AB-Cy7.5** had diffused from the injection site throughout the imageable, shaved region. Taken together, these results suggest that at physiological temperature (∼37 °C), **ABC-Cy7.5** forms a physical hydrogel that remains at the injection site while its diblock counterpart does not, suggesting that the former material could be particularly useful for local drug delivery applications.

## Biocompatibility and drug loading

We have previously reported on soluble PEG-based bottlebrush polymers prepared *via* ROMP;^51,52^ these materials have displayed excellent safety profiles, including in liver and kidney function panels, following systemic administration in mice, rats and dogs. Here, in preparation for *in vivo* local drug delivery studies, we investigated the *in vitro* toxicity and drug release properties of our **ABC** hydrogels. Polymeric amphiphiles are often employed to permeabilize cell membranes;^53^ thus, it was important to determine if **ABC** causes cell membrane damage that could trigger an undesired inflammatory response. Aqueous solutions of **ABC** were incubated with human umbilical vein endothelial cells (HUVEC) for 48 h and the cell culture media was assayed for free lactate dehydrogenase (LDH), which is typically only found in the cytosol of healthy cells and thus represents a biomarker for cell membrane integrity. Polyethylenimine (PEI), a cationic polymer that destabilizes cell membranes, and cell lysate were used as positive controls. Gratifyingly, cells incubated with **ABC** released negligible amounts of LDH, comparable to the levels detected for untreated cells (Fig. S15†), suggesting that **ABC** does not induce rupture of HUVEC cells.

Next, we examined the encapsulation and release of seven small-molecule drugs (dexamethasone, ibuprofen, ofloxacin, SN-38, paclitaxel, gemcitabine, and R848) and a model ∼45 kDa protein (fluorescently labeled ovalbumin, OVA) from **ABC** hydrogels. A straightforward drug encapsulation protocol was developed, where known amounts of dry **ABC** and a given drug were dissolved in dimethyl sulfoxide (DMSO) and then slowly diluted with saline buffer to obtain an aqueous solution with ≤1% organic solvent. The drug-loaded micellar solutions were then spin-concentrated to a TBC concentration of ∼10 wt% where they behaved as free-flowing fluids at room temperature. The drug encapsulation efficiency was determined using liquid chromatography-mass spectrometry (LC-MS) (Table 2 and Fig. S1†). Importantly, the drug-loaded **ABC** solutions retained their ability to undergo rapid gelation upon warming to 37 °C. The extent of drug release *versus* time at 37 °C was assessed by placing the drug-loaded **ABC** hydrogel inside a dialysis membrane (50 kDa MWCO) and measuring the amount of free drug in the exterior saline solution *via* LC-MS for small molecules or UV-vis for OVA (Fig. S16†).

While the encapsulation efficiencies of the individual drugs somewhat correlated with their lipophilicity values (log *D*, Fig. S1†), *i.e.*, the more hydrophobic drugs were encapsulated more efficiently, release was comparable for all drugs—linear release profiles with apparent half-lives of ∼3–5 days were observed. Gratifyingly, **ABC** hydrogel also provided sustained release of the model protein payload, OVA-FITC, suggesting potential applications of this platform for improving the therapeutic index of cytokines and checkpoint inhibitors *via* prolonged intratumor retention. While we note that these release rates could perhaps be extended by altering the composition of **ABC**, our results suggest that the presently studied **ABC** hydrogels are capable of encapsulating and providing controlled release of a range of drugs with varying molecular structures and pharmacological properties.

## Immunochemotherapy studies

Having established that **ABC** hydrogels achieve sustained release of drugs at physiological temperatures *in vitro*, we decided to evaluate the ability of this material to facilitate local release of therapies that activate the immune system. The immune system has been implicated in the efficacy of chemotherapeutics,^54–56^ lending to potential synergy when chemotherapy is combined with additional immune activation. For example, PTX, a canonical microtubule inhibitor, has been shown to enhance DC maturation, cross-presentation of antigen, depletion of myeloid-derived suppressor cells, and tumor penetration by cytotoxic T cells and natural killer (NK) cells—all factors contributing to its anti-tumor effects.^57–60^ Given the immunomodulatory effects of chemotherapy and the release of tumor cell antigens following chemotherapy-induced cell death, it has long been reasoned that combinations of chemotherapy and immunomodulators like TLR agonists may display improved tumor efficacy.^61–63^ Unfortunately, when administered systemically, TLR agonists can induce dramatic flu-like symptoms and related short and long-term side-effects collectively described as cytokine release syndrome.^64,65^ To minimize systemic side effects, drugs with immune-activating mechanisms of action are often administrated locally directly into the accessible tumors.^66,67^ Intratumoral administration, however, does not completely prevent the systemic dissemination of small molecule TLR agonists that can rapidly drain into the vasculature due to their small molecular sizes and the high interstitial pressure within tumors.^68^ Additionally, repetitive administration of high doses of immunomodulators can lead to TLR-tolerance,^69–71^ a phenomenon that might explain tachyphylaxis and the limited effectiveness of free TLR7/8 ligands in early human trials.^64,65,72^ Thus, sustained local delivery is a promising approach to balance the efficacy and side-effects of TLR agonists. To prevent adverse events caused by systemic exposure and to overcome limited efficacy in clinical immunotherapy, research labs both in academia and pharmaceutical companies are exploring ways to achieve prolonged retention of immunomodulators, such as the use of PEG-^73^ or lipid conjugation,^46,72^ and aluminum oxide adsorption.^74^

We reasoned that the **ABC** hydrogel could deliver a combination of R848 and PTX in a sustained fashion, producing a localized anti-tumor immune response while at the same time reducing whole body exposure to these potent molecules. R848 and PTX were chosen based on work from Linnebacher and coworkers showing the superior activity of this combination against colon cancer compared to the first-line medication irinotecan.^75^ Taking advantage of the straightforward drug formulation protocol described above, we loaded **ABC** micelles with a mixture of R848 and PTX and evaluated the efficacy of the resulting material in balb/c mice (*n* = 10 per group) bearing subcutaneous CT26 colon cancer tumors on their hind flank. The CT26 model is a syngeneic tumor model widely used for testing of immunotherapies in immunocompetent mice. We note that all animal studies were conducted under federal, state, and local guidelines in accordance with the Public Health Service Policy on Humane Care and Use of Laboratory Animals and the National Institutes of Health Guide for the Care and Use of Laboratory Animals with approval from the Massachusetts Institute of Technology Committee on Animal Care.

Two different **ABC** micelles with encapsulated PTX and different amounts of R848: **ABC-1** (1.5 mg kg^−1^ of R848 + 10 mg kg^−1^ of PTX) and **ABC-2** (3 mg kg^−1^ of R848 + 10 mg kg^−1^ of PTX), were injected intratumorally every five days for 10 d (3 total injections); tumor volumes and mouse survival were plotted as a function of time for up to 75 d, while the “study readout,” taken as the last day of survival of mice treated with saline as a control, was 48 d. Additional control groups included mice given non-drug-loaded **AB** (“**AB** blank”), non-drug-loaded **ABC** (“**ABC** blank”), the 1× dose PTX and R848 used above but in 75% DMSO/PBS (“free R484 + PTX”), and 1× dose of PTX and R848 used above but in the non-gelling diblock copolymer **AB** (“**AB-1**”).

**Fig. 5:**
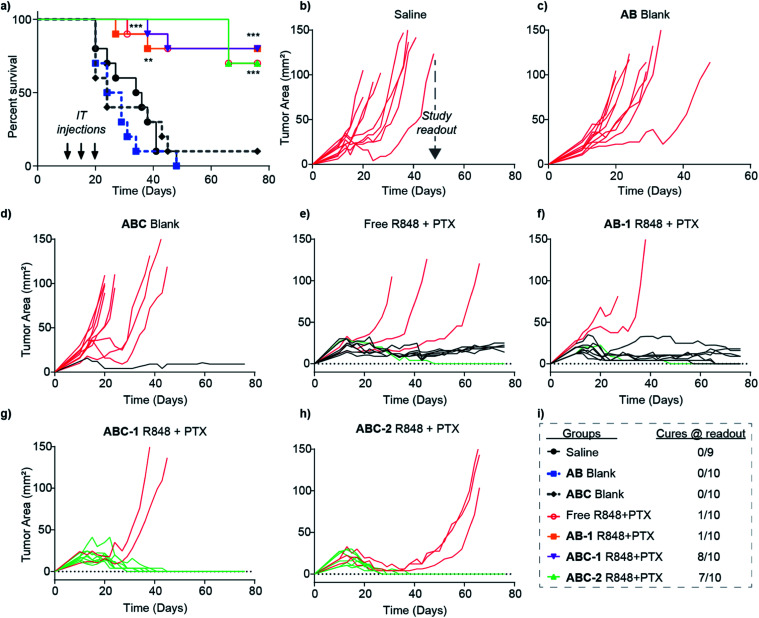
Survival (a) and CT26 tumor growth (b–h) in treatment groups of balb/c mice (*n* = 10). Mice were intratumorally administered R848 at 1.5 (**1**) or 3 mg mL^−1^ (**2**) in combination with 10 mg kg^−1^ PTX formulated in **ABC**, **AB**, or DMSO/saline. Control groups received equal volumes of PBS or blank bottlebrush polymer solutions. Mice in groups are color-coded by non-responders (red lines), limited responders (black lines), and cures (green lines). (i) Efficacy endpoint based on the time of complete loss of the saline control group to the tumor burden. Statistical significance was determined using log-rank (Mantel Cox) test, differences amongst treatment groups compared to saline group have been denoted with ** for *p* < 0.005 and with *** for *p* < 0.0005.

While the PTX + R848 combination conferred a clear survival advantage over saline, **AB** blank, and **ABC** blank, differences between the four treatment groups (free drugs, **AB-1**, **ABC-1**, and **ABC-2**) were not apparent from the average survival curve alone (Fig. 5a), which cannot distinguish between animals that display delayed tumor growth *versus* cures. To highlight differences between these groups at the study readout time (48 d), plots of tumor area for each mouse (Fig. 5b–h) were color-coded by non-responders (red lines), limited responders (black lines), and cures (green lines). From these plots, it is clear that the tumors in mice from the saline (Fig. 5b), **AB** blank (Fig. 5c), and **ABC** blank (Fig. 5d) groups all grew at roughly equal rates with the exception one limited responder from the **ABC** blank cohort. Additionally, while both the free drug (Fig. 5e) and **AB-1** (Fig. 5f) groups displayed improved efficacy, they were dominated by non-responders (3 and 2, respectively) and limited responders (6 and 7, respectively) with only 1 cure per group. In sharp contrast, the **ABC-1** (Fig. 5g) and **ABC-2** (Fig. 5h) groups had 8 and 7 cures (Fig. 5i), respectively, suggesting that the hydrogel-mediated local delivery strategy confers an efficacy advantage through an increase in the number of mice cured per group.

Less frequent, fractionated dosing of TLR7/8 agonist over several hours was found to avert the onset of systemic TLR-tolerance, improving anticancer efficacy of the immunomodulator relative to its bolus injections.^70^ Therefore, it is reasonable that slow-releasing **ABC** formulations elicit faster antitumor activity by providing controlled generation of tumor antigens by PTX synchronized with sustained local activation of innate immune cells by R848.^76^ Properly timed activation of innate immune cells, including dendritic cells, leads to the processing and presentation of tumor-derived antigens to T cells, generating a potent tumor-specific immune response capable of fast eradication of tumors and the potential for long-lasting memory against tumor recurrence. Indeed, it was found that mice cured from the initial **ABC-1** therapy had acquired immune memory against CT26 tumors: long-term surviving mice from the previous efficacy study (*n* = 5) were re-challenged with CT26 tumor cells on their opposite hind flank. Over the course of several weeks after cell implantation, no tumor growth was observed; all tumors were rejected (Fig. S18a†). As a control, naïve mice were inoculated with the same number of cells; rapid tumor outgrowth occurred. In a separate study, groups of mice (*n* = 5 animals per group) were treated intratumorally with only R848 formulated in **ABC** or as free drug solution. Three weeks after a single injection the presence of circulating tumor antigen-specific CD8^+^ T cells was confirmed (Fig. S17b†), further corroborating the induction of tumor-specific immune responses *via* intratumoral injection of R848. Remarkably, the levels of antigen-specific CD8^+^ T cells trended slightly higher in mice treated with R848 formulated in **ABC** compared to free or **AB** formulated R848, though all treated mice had elevated tumor-specific T cell levels in circulation.

**Fig. 6:**
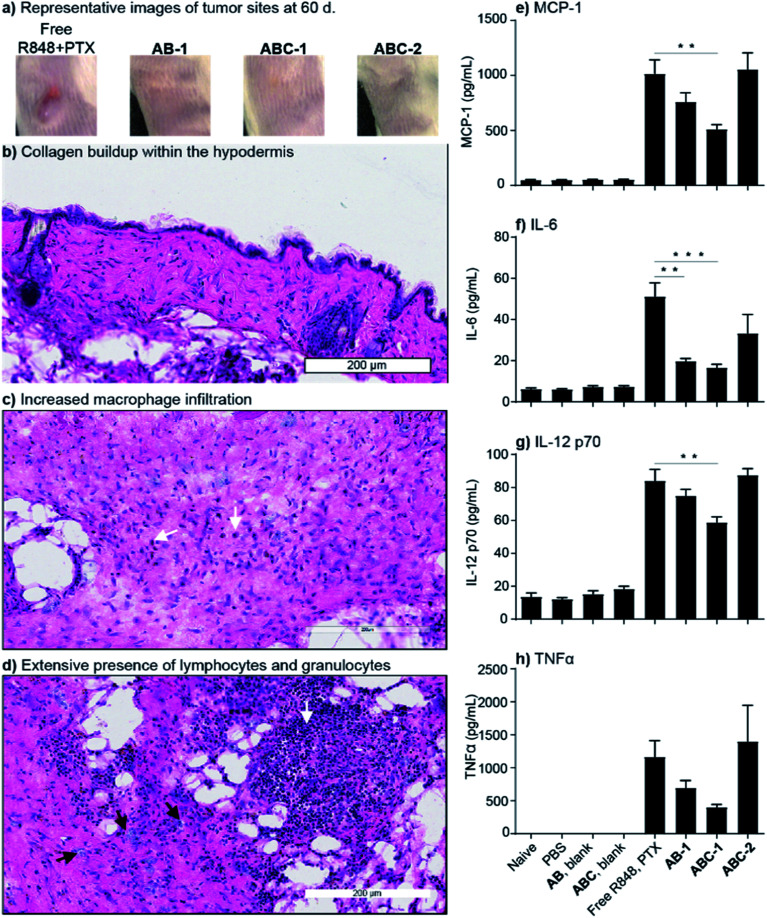
(a) Representative images of tumor sites in treatment groups at 60 days. Histology of injection site in a mouse in the free drugs treatment group showing signs of chronic inflammation (b) collagen under intact dermis, (c) heightened macrophage infiltration, (d) and extensive presences of lymphocytes and granulocytes. Serum cytokine levels in mice groups (*n* = 10) 6 hours after intratumoral injection of formulations of R848 and PTX: (e) monocyte chemoattractant protein-1, MCP-1; (f) Interleukin 6, IL-6; (g) Interleukin 12, IL-12p70; (h) tumor necrosis factor, TNFα. Statistical significance was determined using one-way ANOVA followed by *post hoc* Dunnett's multiple comparisons test, differences amongst treatment groups compared to free R848, PTX group; ** for *p* < 0.005 and with *** for *p* < 0.0005.

In addition to the improved efficacy observed above for **ABC** hydrogel mediated R848 delivery, we noted visible inflammation at the site of injection of the free drug combination that was not present for the **ABC** hydrogel groups (Fig. 6a and S18†). This observation suggested that without **ABC** hydrogel, the free drug combination induces local inflammation that takes considerably longer to clear. Histological analyses of tissue samples from the group administered the free drug combination were conducted to assay for signs of chronic inflammation associated with topically applied immune stimulators.^77,78^ As shown in Fig. 6a these mice showed signs of chronic inflammation that still persisted at 60 d, while the tumors were completely cured in the **ABC** treatment groups (Fig. S18†). Despite the intact epidermis and dermis, histological analysis revealed collagen buildup within the hypodermis of the free-drug-treated mice, indicative of scar tissue formation (Fig. 6b–d and S19a†). Moreover, extensive macrophage infiltration was noted beneath the skin (Fig. 6c), while lymphocyte and granulocyte accumulation was also present at the site of the scar (Fig. 6d). Meanwhile, no visible signs of chronic inflammation were detected in the **ABC** hydrogel groups (Fig. S18†).

To assay for undesired systemic inflammation, serum samples collected 6 h after the first intratumoral injection were assayed for four cytokines—MCP-1, IL-12, IL-6, and TNF—that are produced by dendritic cells and macrophages upon TLR-7/8 activation and thus serve as biomarkers for systemic immune activation (Fig. 6e–h). In support of our hypothesis, 2–3-fold lower levels of MCP-1, IL-12, and IL-6 were measured for the **ABC-1** group compared to the free drug group. Moreover, the **ABC-2** group, which received twice the amount of R848 overall, displayed similar cytokine levels as the 1× free drug group, showing that the local delivery strategy can facilitate the use of higher doses with comparable systemic side effects. Interestingly, even the **AB**-encapsulated PTX-R848 combination led to lower amounts of systemic cytokines compared to the free drug group, likely due to the lower bioavailability of the encapsulated drug. As expected, no increased cytokine production was detected in the control groups. Altogether, these data show the advantage of our TBC hydrogel for local R848 and PTX delivery, leading to both an increase in efficacy (Fig. 5) and improved safety (Fig. 6), which translate to an overall improved therapeutic index.

The reduction of systemic cytokines using **ABC** hydrogels is likely due to prevention of burst exposure of R848 (Fig S14 and S16†). These results align with a pharmaceutical industry-wide shift toward immunomodulator formulations that retain the drug at the site of injection, thus enhancing therapeutic index.^72–74^ In addition to induction of higher unwanted systemic inflammatory cytokines, intratumoral injections of free R848 + PTX resulted in delayed cures with persistent inflammation at the injection site, which are likely caused by burst exposure to PTX with subsequent massive antigen release occurring after a majority of the R848 immunomodulator has diffused away from the site of injection, promoting tolerance.^56,79^ Indeed, it has been previously demonstrated that improper timing of immune activation and antigen release can significantly dampen antitumor immune responses.^80^ We also observed higher systemic levels of the anti-inflammatory cytokine IL-10 for longer periods in mice given the free drug combination (Fig. S19b†), which could partially explain delayed clearing of the tumors.^81^

The narrow therapeutic indices of systemically administered traditional chemotherapeutics give rise to their well-known side effects. Similar challenges are surfacing in the promising field of immunochemotherapy, motivating the development of intratumoral delivery strategies. Wide adoption of such combination therapies, however, demands the availability of smart vehicles for local drug delivery that are capable of maintaining optimal concentrations of chemotherapeutics and immunodulators in tumor tissue to achieve curing effects without producing tolerogenic or unwanted side effects. Our results demonstrate that TBC-based hydrogels enable delivery of a combination of a chemotherapeutic (PTX) and a TLR7/8 agonist (R848), promoting tumor-controlling immunologic effects while reducing dose-limiting systemic and local toxicities associated with such therapies.

## Conclusions

We developed an effective protocol for the synthesis of modular TBCs. Using a range of physicochemical characterization techniques, we demonstrate that “ABC” sequenced TBCs self-assemble into well-defined stable micelles at room temperature, allowing for efficient encapsulation of variety of bioactive payloads. We further show that at physiological temperatures (*T* > 32 °C), aqueous solutions of **ABC** TBCs undergo rapid gelation, forming a localized depot for sustained release of encapsulated therapeutics. These TBC amphiphiles represent a universal platform for local drug delivery, with efficient drug encapsulation, straightforward formulation and administration, and the ability to provide sustained release for both small molecule drugs and macromolecular payloads, properties that bode well for future applications in areas ranging from anticancer chemo- and immunotherapy to ophthalmic therapy.

## Abbreviations

PLA: Polylactic acid
PEG: Polyethylene glycol
PNIPAM: Poly(*N*-isopropylacrylamide)
LCST: Lower critical solution temperature
*T*_g_: Glass transition temperature
ROMP: Ring-opening metathesis polymerization

## Author contributions

The manuscript was written through contributions of all authors. All authors have given approval to the final version of the manuscript.

## Funding sources

JAJ acknowledges support from the National Institutes of Health (1R01CA220468-01) and London Eye Hospital Pharma. LEM is supported by the National Institutes of Health NIGMS Interdepartmental Biotechnology Training Program (T32-GM008334). This work was supported in part by the Marble Center for Nanomedicine and the Koch Institute Support (core) Grant P30-CA14051. DJI is an investigator of the Howard Hughes Medical Institute.

## Conflicts of interest

There are no conflicts of interest to declare.

## Supplementary Material

SC-011-D0SC02611E-s001
