# Supplementary material for: ABC triblock bottlebrush copolymer-based injectable hydrogels: design, synthesis, and application to expanding the therapeutic index of cancer immunochemotherapy
Source: Chem Sci. 2020 Jun 1;11(23):5974–86. doi: 10.1039/d0sc02611e (PMC8159417; doi:10.1039/d0sc02611e)
Supplement: SC-011-D0SC02611E-s001 [file SC-011-D0SC02611E-s001.pdf]

Electronic Supplementary Material (ESI) for Chemical Science.
This journal is © The Royal Society of Chemistry 2020

Supporting information for:

ABC triblock bottlebrush copolymer-based injectable hydrogels: design, synthesis, and application to expanding the therapeutic index of cancer immunochemotherapy

Farrukh Vohidov,[1,‡] Lauren E. Milling,[2,‡] Qixian Chen,[1] Wenxu Zhang,[1] Sachin Bhagchandani,[1] Hung, V.-T. Nguyen,[1] Darrel J. Irvine,[2] Jeremiah A. Johnson,[1,2]**

[1]Department of Chemistry, Massachusetts Institute of Technology, Massachusetts 02139, United States

[2]Koch Institute for Integrative Cancer Research, Massachusetts Institute of Technology, Cambridge, Massachusetts 02139, United States

[‡] These authors contributed equally to this work.

Contents

1. **General information** 3
2. **Synthetic procedures** 10
   2.1. Norbornene-[poly(N-isopropylacrylamide] macromonomer (Nb-PNIPAM MM). 10
   2.2. Norbornene-(polylactic acid) macromonomer (Nb-PLA MM). 10
   2.2. Sequential ROMP for synthesis of AB bottlebrush block copolymer. 11
   2.3. Sequential ROMP for synthesis of ABC bottlebrush block copolymer. 12
3. **Supplemental figures** 14
4. **Supplemental spectra** 31
5. **References** 38

1. General information

All reagents were purchased from commercial suppliers and used without further purification unless stated otherwise. Ibuprofen, dexamethasone, oflaxacin, DL-lactide, NH_2-PEG (3 KDa) and NH_2-PNIPAM (5.5 KDa) were purchased from Sigma. Resiquimod was purchased from Selleckchem. Paclitaxel and SN-38 were purchased from Ark Pharma. Gemcitabine was purchased from Santa Cruz Biotechnology. Ovalbumin, Alexa Fluor™ 647 Conjugate was purchased from ThermoFisher scientific.

Preparation of known compounds and reagents. Grubbs' 3rd generation bispyridyl catalyst,[1] norbornene-*N*-hydroxysuccinimide,[2] norbonere alcohol,[3] **Nb-PEG**[2] and **Nb-PEG Cy7.5 MM**[4] were prepared according to literature procedures.

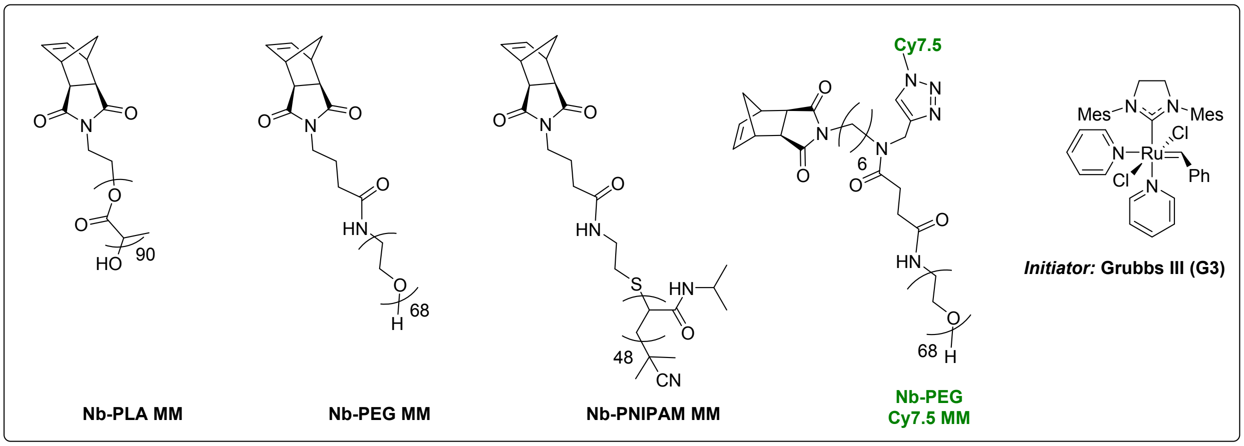

Nuclear Magnetic Resonance. ^{1}H NMR spectra were measured on a Bruker 400 MHz or a 500 MHz spectrometer. All ^{13}C NMR were recorded at 125 MHz. All purified products were determined to be ≥95% pure unless otherwise noted.

Mass spectrometry. MALDI-TOF MS were performed on a Bruker OmniFlex mass spectrometer with Sinapic acid matrix (Fluka, 20 mg/mL). ESI-MS was performed on a Bruker Daltonics micrOTOF instrument.

Liquid Chromatography Mass Spectrometry (LC-MS). LC-MS was performed on an Agilent 1260 Infinity II LC with a quaternary pump, autosampler, column thermostat, diode array detector, and an Agilent 6120B mass spectrometer. The optimized mobile phase consisted of 0.1% formic acid in acetonitrile and water using 1 mL/min flow rate. LC parameters: 5 µl injection, Agilent Zorbax Eclipse XDB 4.6 x 150 mm column with 2.7 µm particles, maintained at 35 °C; 242 nm; 10% - 50% MeCN/H_2O [1 – 3 min]; 95% - 100% MeCN/H_2O [6 – 7 min].

Preparative Size Exclusion Chromatography. Preparative SEC was performed on a recycling LaboACE system (Japan Analytical Industry) using JAIGEL-2.5HR column.

Analytical Size Exclusion Chromatography. Brush samples were analyzed using an Agilent 1260 Infinity setup with two Shodex KD-806M columns in tandem and a 0.025 M LiBr DMF mobile phase run at 60 °C. The differential refractive index (dRI) was monitored using a Wyatt Optilab T-rEX detector.

Rheology. Measurements were performed on a TA Instruments Discovery HR-2 rheometer using an 8-mm parallel plate geometry. Around 0.4 mL of the TNB aq. solutions were placed and upper arm lowered to 1100 µm, achieving flush contact between sample and the geometry. Instrument's sample covers were used to minimize evaporation during

measurements. Frequency of 10 rad/s and a heating rate of 1 °C/min were used to assay the temperature dependence of G' and G''.

Dynamic Light Scattering. Measurements were performed using a Wyatt Technology Mobius DLS instrument. Samples were prepared at 1.0 mg/mL in either nanopure water (MilliQ) or PBS buffer, the samples were filtered through a 0.4 μm Nalgene filter (nylon membrane) into disposable polystyrene cuvettes, which were pre-cleaned with compressed air. Measurements were made in sets of 10 acquisitions, and the average hydrodynamic diameters were calculated using the DLS correlation function via a regularization fitting method (Dynamics 7.4.0.72 software package from Wyatt Technology).

Transmission Electron Microscopy. TEM images were acquired using a FEI Tecnai Multipurpose TEM (G2 Spirit TWIN, 12kV). Dilute (≥1.0 mg/mL) aqueous solutions of brush amphiphiles were pipetted onto a carbon film-coated 200-mesh copper grid (Electron Microscopy Sciences) placed on a piece of parafilm. The samples were let to sit for 5 minutes and then the solution was carefully absorbed at the base of the droplet using the edge of a Kimwipe, leaving behind the nanoparticles on the TEM grid. The samples were then stained negatively by adding a drop of 2 wt% uranyl acetate (Electronic Microscopy Sciences). After 3 min, the residual uranyl acetate solution was carefully absorbed onto a Kimwipe, and the samples were allowed to dry completely before analysis. thickness: 1 mm).

Cryo-TEM. Aqueous solution of bottlebrush micelles (3 μL of 1 wt%) was dropped on a lacey copper grid coated with a continuous carbon film and blotted to remove excess

sample without damaging the carbon layer by Gatan Cryo Plunge III. Grid was mounted on a Gatan 626 single tilt cryo-holder equipped in the TEM column. The specimen and holder tip were cooled down using liquid-nitrogen, maintaining ethane-frozen sample at low temperatures during transfer into the microscope and subsequent imaging. Imaging on a JEOL 2100 FEG microscope was done using minimum dose method to avoid sample damage under the electron beam. The microscope was operated at 200 kV and with a magnification in the ranges of 10,000~60,000 for assessing particle size and distribution. All images were recorded on a Gatan 2kx2k UltraScan CCD camera.

Small Angle X-ray Scattering (SAXS): Scattering data were collected at beamline 12-ID-B at the Advanced Photonic Source (APS) at Argonne National Laboratory. The energy of the beam was 14 keV, which corresponds to a wavelength of 0.08857 nm. The system was calibrated using a silver behenate standard. Variable-temperature (VT) experiments were conducted using in situ heating on a Linkam temperature control stage. All SAXS samples were prepared by loading aqueous solutions of TBCs loaded into the center of Bokers aluminum washers (0.900 ± 0.005" OD x 0.079 ± 0.005" ID) with 0.40 ± 0.04" thickness and sealing both sides with Kapton tape.

In vitro release assay. R848 (120 μg)/PTX (200 μg) loaded **ABC** hydrogels (~100 mg/ml, 50 μl) were placed in dialysis tubing (8-10 kD molecular weight cut-off) and suspended in PBS (950 μl). The dialysis bags were loaded into 50-mL conical tubes containing 25 ml sink medium (PBS), and incubated at 37°C with a shaking speed of 125 rpm. The sink medium (1 mL) was removed and replenished with the same volume of fresh, prewarmed

PBS, thus ensuring constant sink conditions. Aliquots were lyophilized and dissolved in 250 µl methanol, drug content determined using LC-MS.

OVA release: The **ABC** hydrogel in PBS (100 mg/mL, 50 µl) was mixed with solution of fluorescently labeled OVA (2 mg/mL, 5 µl) and vortexed for 1 minute. The resulting solution was heated until gel formation and then 200 µl of pre-warmed PBS was added to the vial, forming an aqueous layer over hydrogel. 100 µl aliquots were taken at defined time-points and sink media was refilled with equal volume of prewarmed PBS. The amount of OVA in aliquots was determined using fluorescence measurements (Ex/Emm = 650/680 nm)

Cell culture: CT26 cell were cultured in RPMI media (ThermoFisher Scientific), supplemented with 10% fetal bovine serum (FBS, VWR) and 1%penicillin/streptomycin (ThermoFisher Scientific). Human umbilical vein endothelial cells (HUVEC, Lonza) were cultured in EGM^+ media (Lonza) supplemented with 1% penicillin/streptomycin. All cells were housed in 5% CO_2 humidified atmosphere at 37 °C.

Lactate dehydrogenase (LDH) assay. HUVEC cells were plated at 10,000 cells per well (in 100 µL) in 96-well collagen coated plates (Corning) and were allowed to adhere overnight. Then, free media containing polymer solutions were added. The plates were incubated for 72 h, aliquots of supernatant were taken, and the levels of LDH were measured using the LDH-Glo™ Cytotoxicity Assay (Promega).

Animal use. All mice were purchased from Jackson lab. Animal studies were conducted under federal, state, and local guidelines in accordance with the Public Health Service Policy on Humane Care and Use of Laboratory Animals and the National Institutes of Health *Guide for the Care and Use of Laboratory Animals* with approval from the Massachusetts Institute of Technology Committee on Animal Care. Protocol number: 0817-081-20.

Whole-mouse imaging. Female Balb/C mice (3 animals/group) were shaved around the left hind flank and anesthetized by inhaled 2-3% isoflurane, and the anesthesia verified by the lack of reaction to toe pinch and breathing rhythm. After cleaning the shaved area with aq. ethanol (70% v/v), using a tweezer the skin over left hind flank was raised and 30 μL of fluorescently labeled **ABC-Cy7.5** or **AB-Cy7.5** solutions (10 wt %) in PBS injected subcutaneously. Retention of **ABC-Cy7.5** and **AB-Cy7.5** at the injection site were monitored using an IVIS fluorescence imaging system. Imaging setting were kept identical between imaging sessions, measured 30 min after injection and again 24 hours later (Ex/Emm = 745/800 nm).

CT26 cancer model. Female Balb/C mice, 7-9 weeks of age, were shaved around the right hind flank and their weights measured (18-20 g body weight). Mice were anesthetized by inhaled 2-3% isoflurane, and the anesthesia verified by the lack of reaction to toe pinch and breathing rhythm. 0.5 mil cells were injected subcutaneously into the right hind flanks. After 10 days, the tumor sizes were measured (digital calipers) and mice were randomized into treatment groups with similar average tumor sizes, and the treatments were started.

Following formulations were prepared at the day of injections: i) **ABC-1** (R848 at 1 mg/mL, PTX at 6.7 mg/mL, 10 mg/mL polymer in PBS); ii) **ABC-2** (R848 at 2 mg/mL, PTX at 6.7 mg/mL, 10 mg/mL polymer in PBS); iii) **AB-1** (R848 at 1 mg/mL, PTX at 6.7 mg/mL, 10 mg/mL polymer in PBS); iv) "free" R848+PTX (R848 at 1 mg/mL, PTX at 6.7 mg/mL in 75% DMSO/PBS v/v solution). Each injection was performed using insulin needles (30-G), 30 µL intratumorally, which is equivalent to 30 µg or 60 µg R848 and 200 µg PTX dose. Injections were performed every 5 days for 15 days. Tumor sizes were measured twice a week, mice with tumor sizes >100 mm^2 were euthanized (CO_2 asphyxiation followed by cervical dislocation). All collected data has been entered, graphed and analyzed using GraphPad Prizm 7.02.

Serum cytokine measurements. The serum samples, collected 6 and 24 h after first injection, were analyzed by cytometric bead array using the CBA Mouse Inflammation Kit from BD biosciences following manufacturer's protocols. Mean fluorescence intensity was then compared to a standard curve for each cytokine.

Tetramer Staining. Blood was acquired by retro-orbital bleeding of mice three weeks after the first injection of drug. Red blood cells were lysed using ACK lysing buffer (ThermoFisher Scientific). Gp70 tetramer (H-2L^d MuLV gp70 Tetramer-SPSYVYHQF-PE) was purchased from MBL. Tetramer staining was performed at room temperature in buffer containing 50-nM dasatinib to enhance staining. Afterwards, anti-mouse CD8α (53-6.7) (Biolegend) was utilized to stain $CD8^+$ T cells. Viability was assessed using DAPI.

Cells were analyzed using BD-FACSCanto flow cytometer, and data were analyzed using FlowJo.

2. Synthetic procedures

2.1. Norbornene-[poly(N-isopropylacrylamide] macromonomer (Nb-PNIPAM MM).

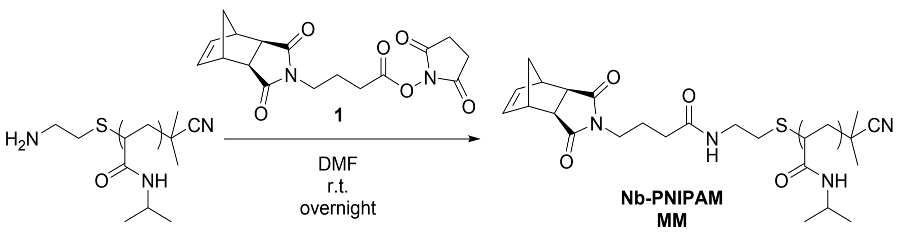

To a 20-mL vial containing amine-terminated poly(N-isopropylacrylamide) (NH_2-PNIMAP) (900 mg, 0.16 mmol) was added norbornene-*N*-hydroxysuccinimide **1** (60.5 mg, 0.17 mmol) followed by anhydrous DMF (6 mL). The resulting mixture was stirred overnight at room temperature. Then the reaction mixture was diluted with chloroform, filtered through a 0.22 μm nylon syringe filter, and loaded onto preparatory SEC column for purification. Isolated as beige solid (747 mg, 83%).

2.2. Norbornene-(polylactic acid) macromonomer (Nb-PLA MM).

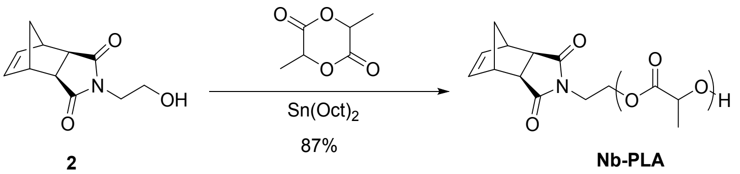

Into a μw-vial was added DL-lactide (2.46 g, 0.017 mol) and norbonere alcohol **2** (88.3 mg, 0.426 mmol), the vial was sealed and then evacuated and backfilled with nitrogen. The vial was placed in oil bath and heated to 130 °C and to the molten lactide was added tin(II) 2-ethylhexanoate (14 μL, 0.027 mmol) under nitrogen. The reaction mixture was stirred

for 30 min and then let to cool down to room temperature. Then the reaction mixture was dissolved in DCM and then precipitated into cold MeOH. This step was repeated three times to remove residual lactide. Dried under vacuum, isolated as sugary white material (2.15 g, 87% yield).

2.2. Sequential ROMP for synthesis of AB bottlebrush block copolymer.

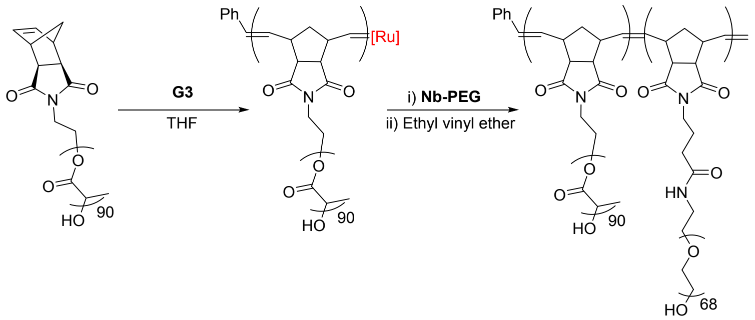

In a nitrogen filled glovebox, to a 4-mL vial charged with a stir bar and containing **Nb-PLA MM** (227 mg, 0.0344 mmol) was added anhydrous THF (750 µL). After dissolution of macromonomer, solution of Grubbs III catalyst was added all at once (165 µL, 0.0045 mmol). The polymerization reaction was stirred at room temperature for 15 min. Then a 10-µL aliquot was taken for size exclusion chromatography analysis and solution of **Nb-PEG MM** in THF (600 mg, 0.1820 mmol) was added to the reaction mixture. After 30 m the reaction mixture was taken out of the glovebox and polymerization quenched by addition of ethyl vinyl ether (100 µL, excess) and allowed to stir for 10 min. A 10-µL aliquot was taken out for size exclusion chromatography analysis of polymerization product, after which the bottlebrush solution was diluted with milliQ water (10 mL) and transferred into 50 kDa MWCO dialysis tubing (Spectrum Laboratories). The solution was dialyzed against milliQ water (16 L×3, water replaced with fresh milliQ water every 2 h). The brush solution

was then transferred into 100 KDa MWCO spin filter and concentrated to ~4 mL. Then diluted with milliQ water and concentrated again to ensure removal of any unconverted macromonomer. Then the brush solution was flash frozen using liquid nitrogen and lyophilized to afford white cottony solid (804 mg, 97% isolated yield). A similar protocol was used for synthesis of **AB-Cy7.5** bottlebrush, where during polymerization of PEG block was added 1% mol of **Nb-PEG Cy7.5 MM**.

2.3. Sequential ROMP for synthesis of ABC bottlebrush block copolymer.

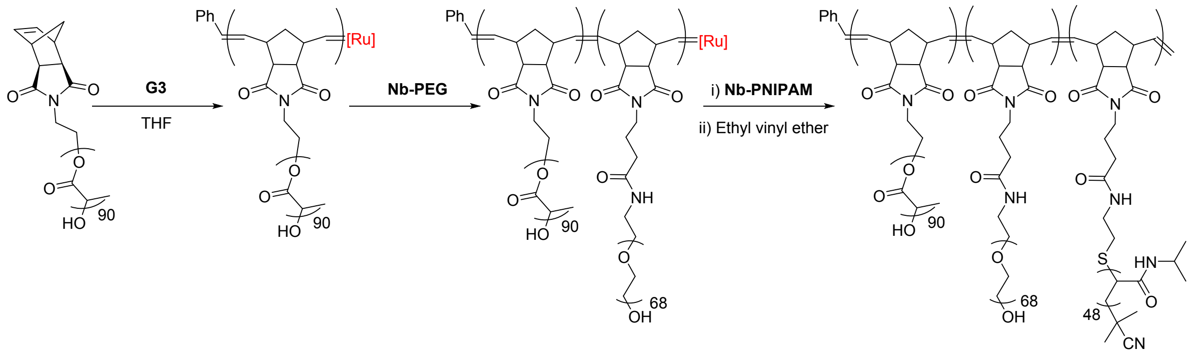

In a nitrogen filled glovebox, to a 4-mL vial charged with a stir bar and containing **Nb-PLA MM** (192 mg, 0.0291 mmol) was added anhydrous THF (600 µL). After dissolution of macromonomer, solution of Grubbs III catalyst was added all at once (140 µL, 0.0038 mmol). The polymerization reaction was stirred at room temperature for 15 min. Then a 10-µL aliquot was taken for size exclusion chromatography analysis and solution of **Nb-PEG MM** in THF (508 mg, 0.1539 mmol) was added to the reaction mixture. After additional 20 min another 10-µL aliquot was taken for analysis and then solution of **Nb-PNIPAM MM** in THF (221 mg, 0.0401 mmol) was added to the reaction mixture. After 1 h the reaction mixture was taken out of the glovebox and polymerization quenched by addition of ethyl vinyl ether (100 µL, excess) and allowed to stir for 10 min. A 10-µL aliquot was

taken out for size exclusion chromatography analysis of polymerization product, after which the bottlebrush solution was diluted with milliQ water (20 mL) and transferred into 50 kDa MWCO dialysis tubing (Spectrum Laboratories). The solution was dialyzed against milliQ water (16 L×3, water replaced with fresh milliQ water every 2 h). The brush solution was then transferred into 100 KDa MWCO spin filter and concentrated to ~4 mL. Then diluted with milliQ water and concentrated again to ensure removal of any unconverted macromonomer. Then the brush solution was flash frozen using liquid nitrogen and lyophilized to afford white cottony solid (530 mg, 57% isolated yield). A similar protocol was used for synthesis of **ABC-Cy7.5** bottlebrush, where during polymerization of PEG block was added 1% mol of **Nb-PEG Cy7.5 MM**.

3. Supplemental figures

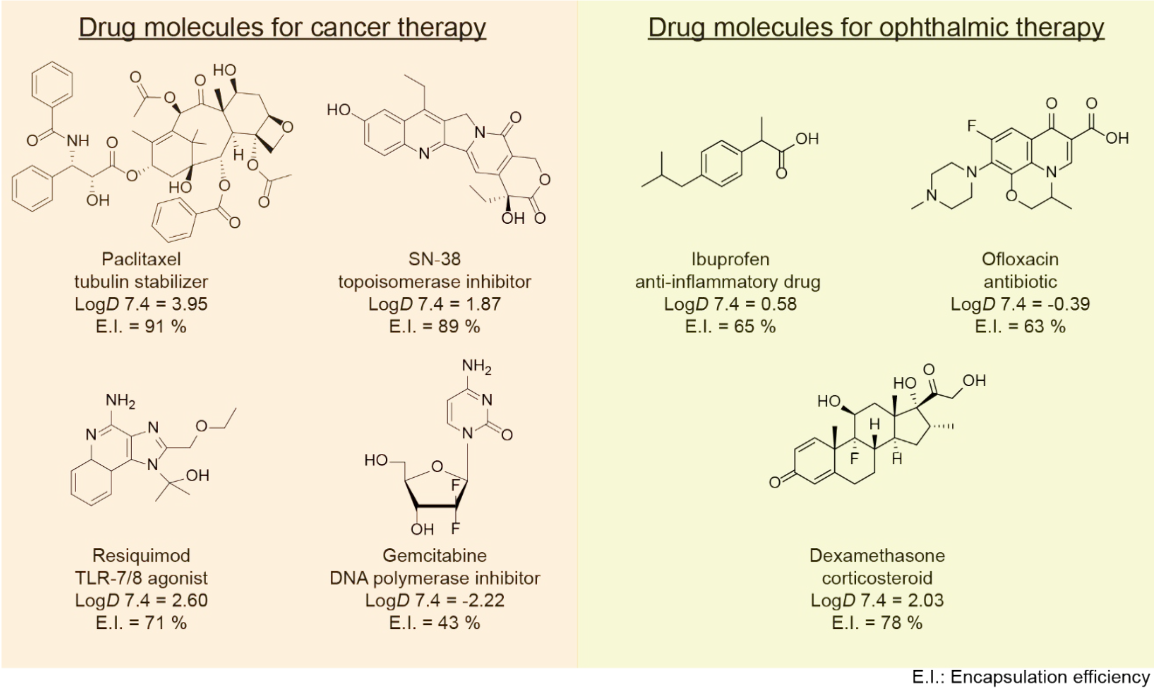

Figure S1. Structures, lipophilicity values, and encapsulation efficiencies of small molecule drugs used for encapsulation studies.

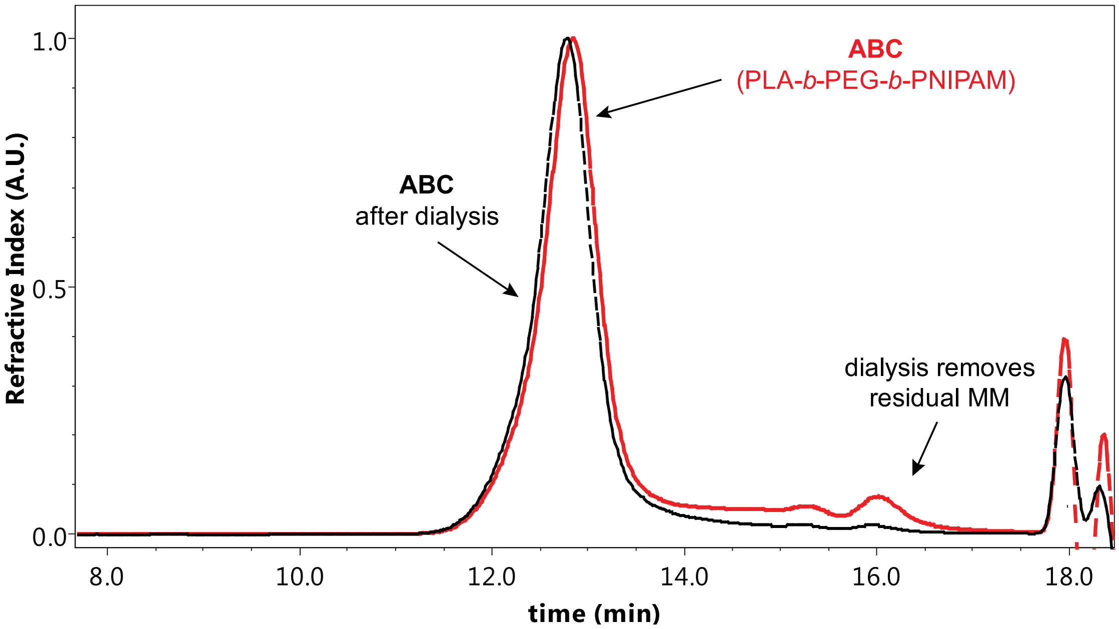

Figure S2. SEC analysis of triblock bottlebrush copolymer **ABC** demonstrating removal of the majority of residual MMs after dialysis.

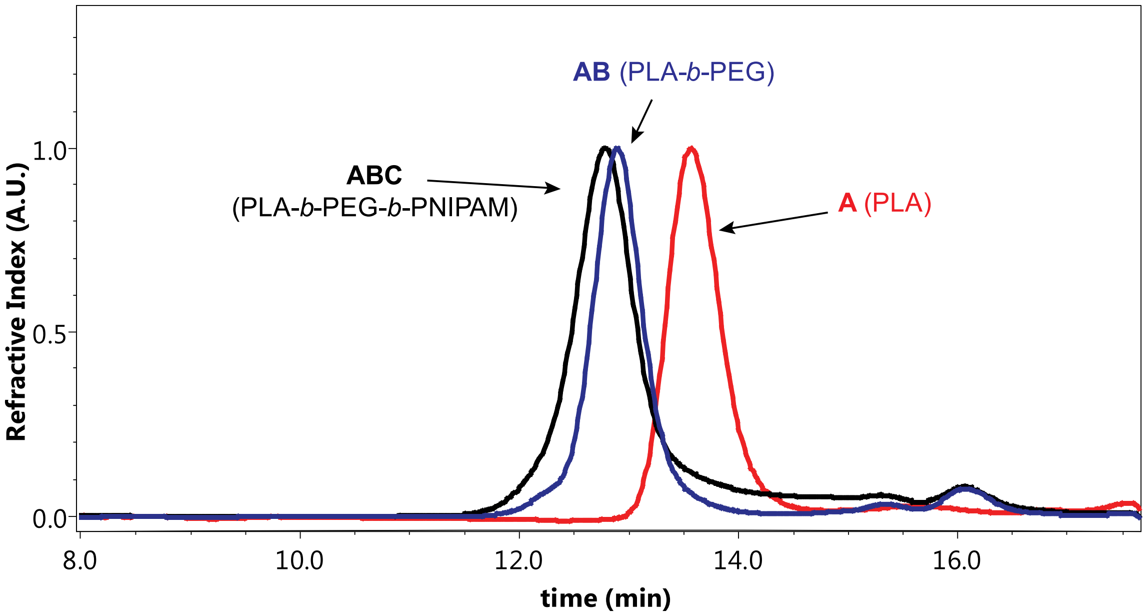

Figure S3. SEC analysis of a typical sequential ROMP of norbornene-MMs. Aliquots taken for SEC analysis after polymerization of each successive block.

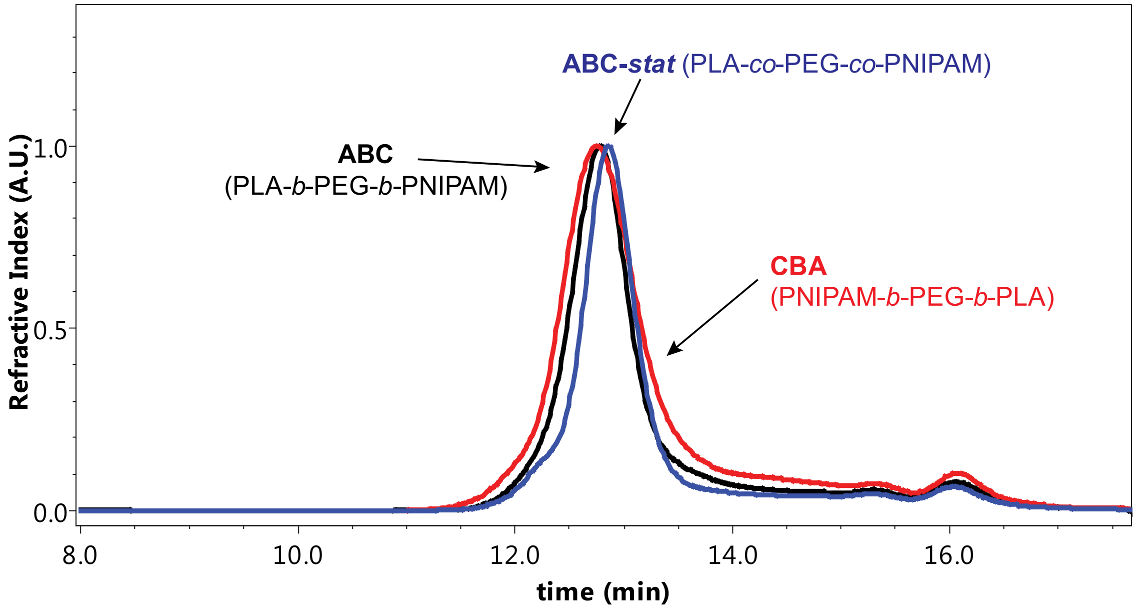

Figure S4. SEC analysis of bottlebrush copolymers **ABC**, **CBA**, and **ABC-*stat*** synthesized using identical MM : initiator ratios, thus having comparable composition.

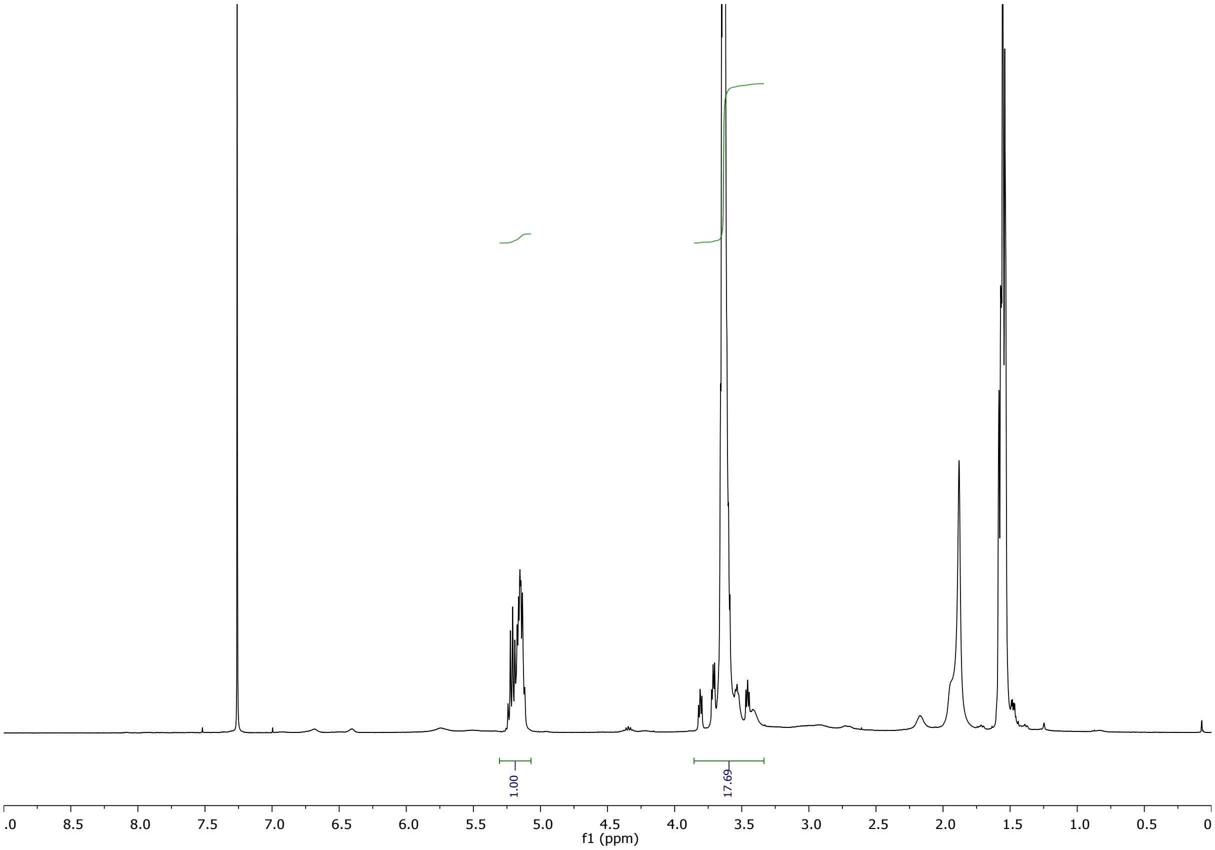

Figure S5. ^{1}H NMR spectrum of **AB** bottlebrush diblock copolymer in $CDCl_3$.

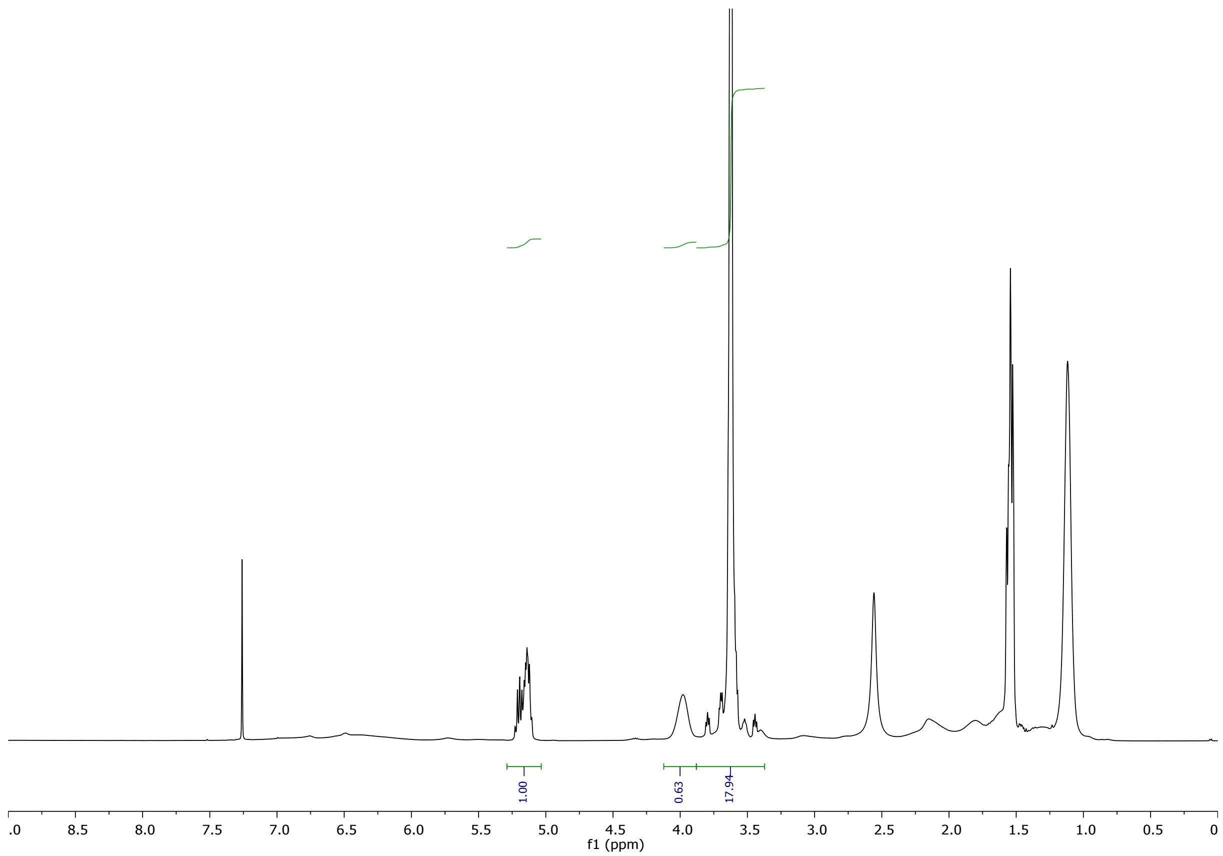

Figure S6. ^{1}H NMR spectrum of **ABC** bottlebrush triblock copolymer in $CDCl_3$.

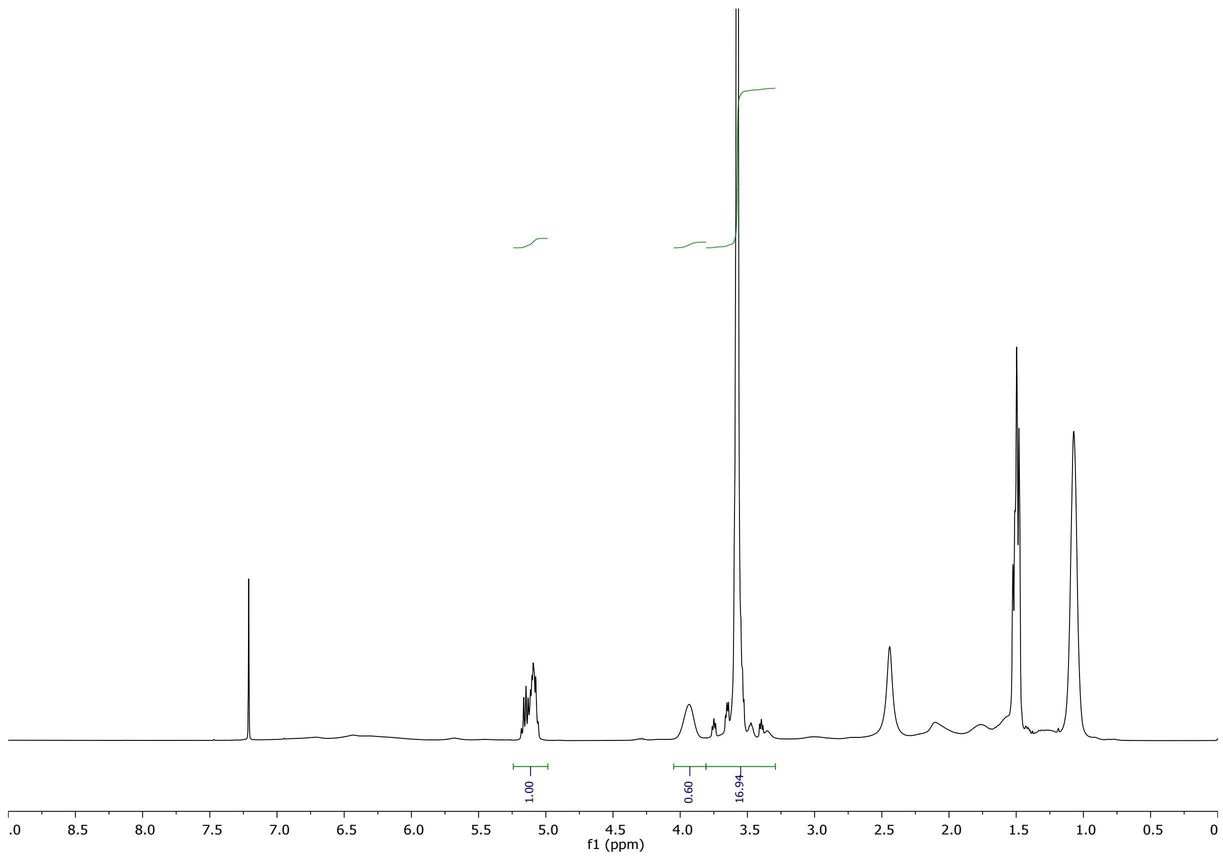

Figure S7. ^{1}H NMR spectrum of **ABC-*stat*** bottlebrush triblock copolymer in $CDCl_3$.

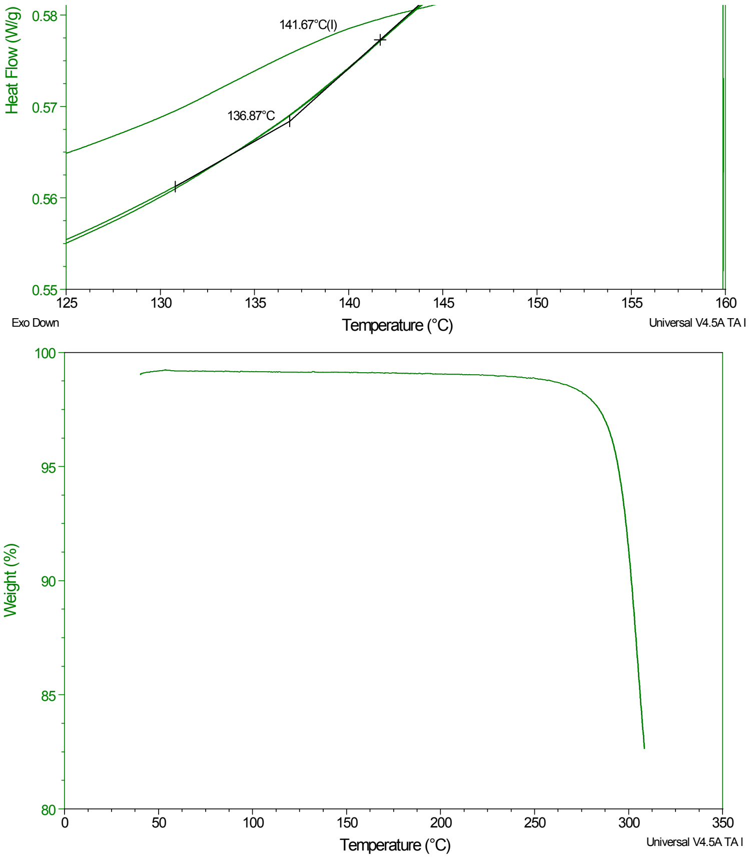

Figure S8. DSC (top, middle) and TGA (bottom) analysis of **ABC**. T_m of PEG and T_g of PNIPAM blocks observed at 50.56 °C and at 141.67 °C, respectively.

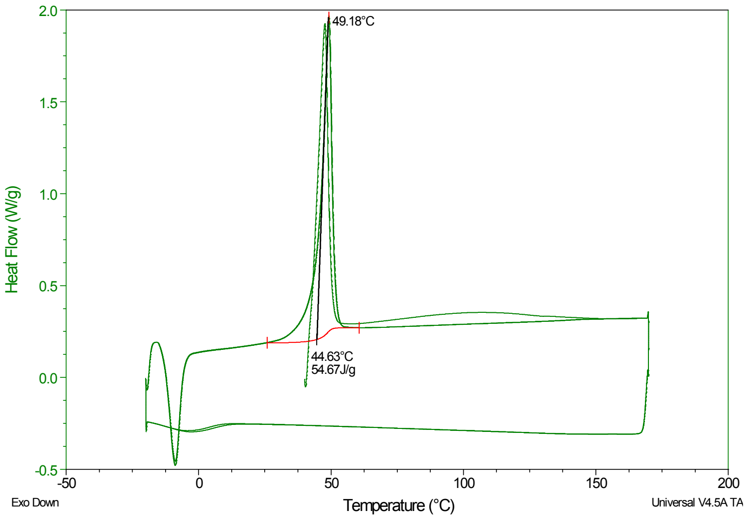

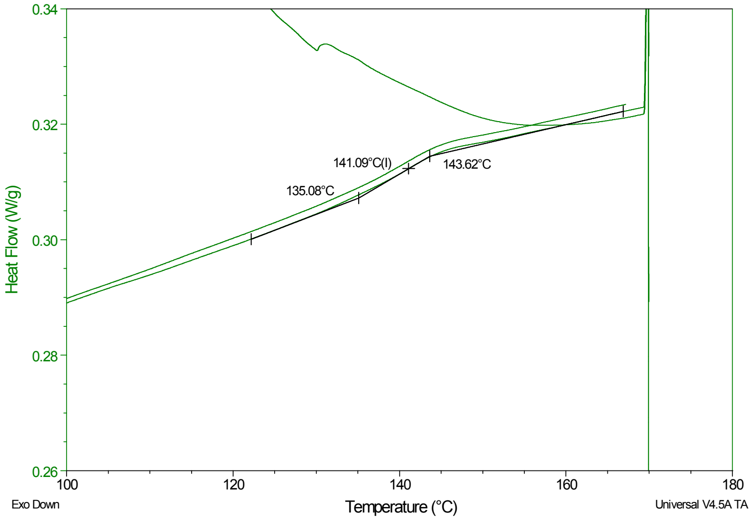

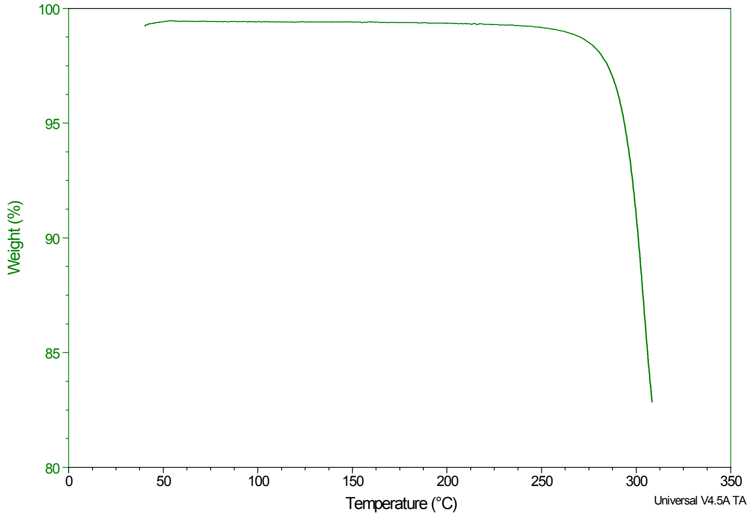

Figure S9. DSC (top, middle) and TGA (bottom) analysis of triblock bottlebrush copolymer **ABC-*stat***. T_m of PEG and T_g of PNIPAM observed at 49.18 °C and at 141.09 °C, respectively.

a) ABC

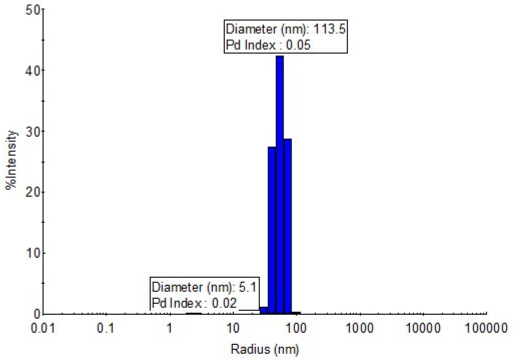

b) CBA

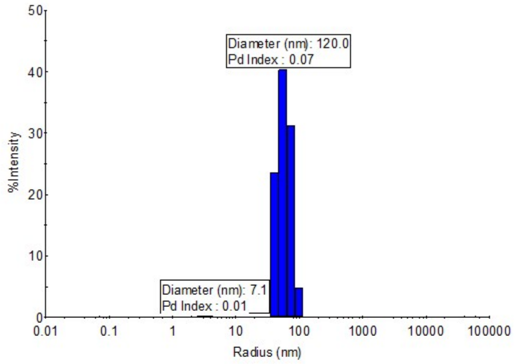

c) ABC-*stat*

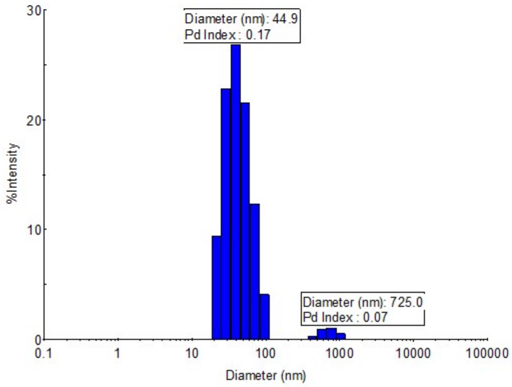

d) AB

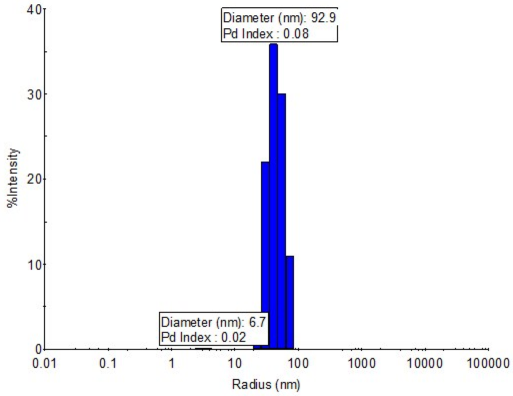

Figure S10. DLS measurements of bottlebrush amphiphiles in water (1 mg / mL). a) **ABC**; b) **CBA**; d) **ABC-*stat***; e) **AB**.

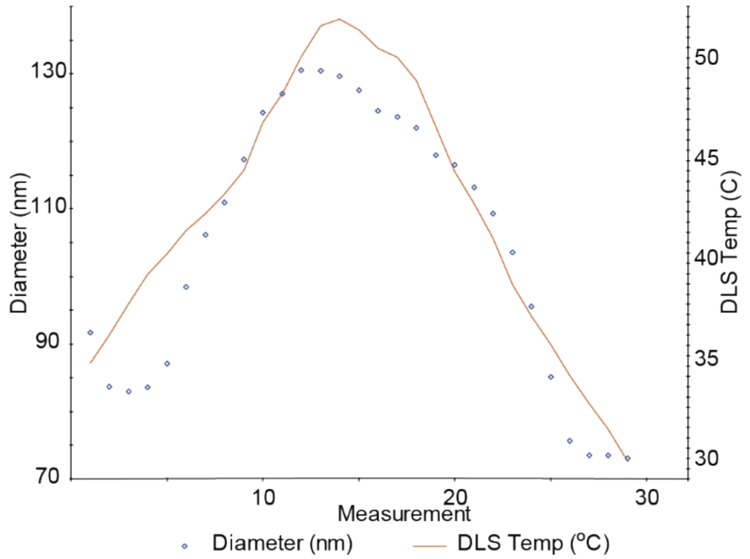

Figure S11. Variable-temperature DLS measurements demonstrate the reversible formation of **ABC** aggregates with increased temperature (samples prepared at 1 mg of polymer / mL water).

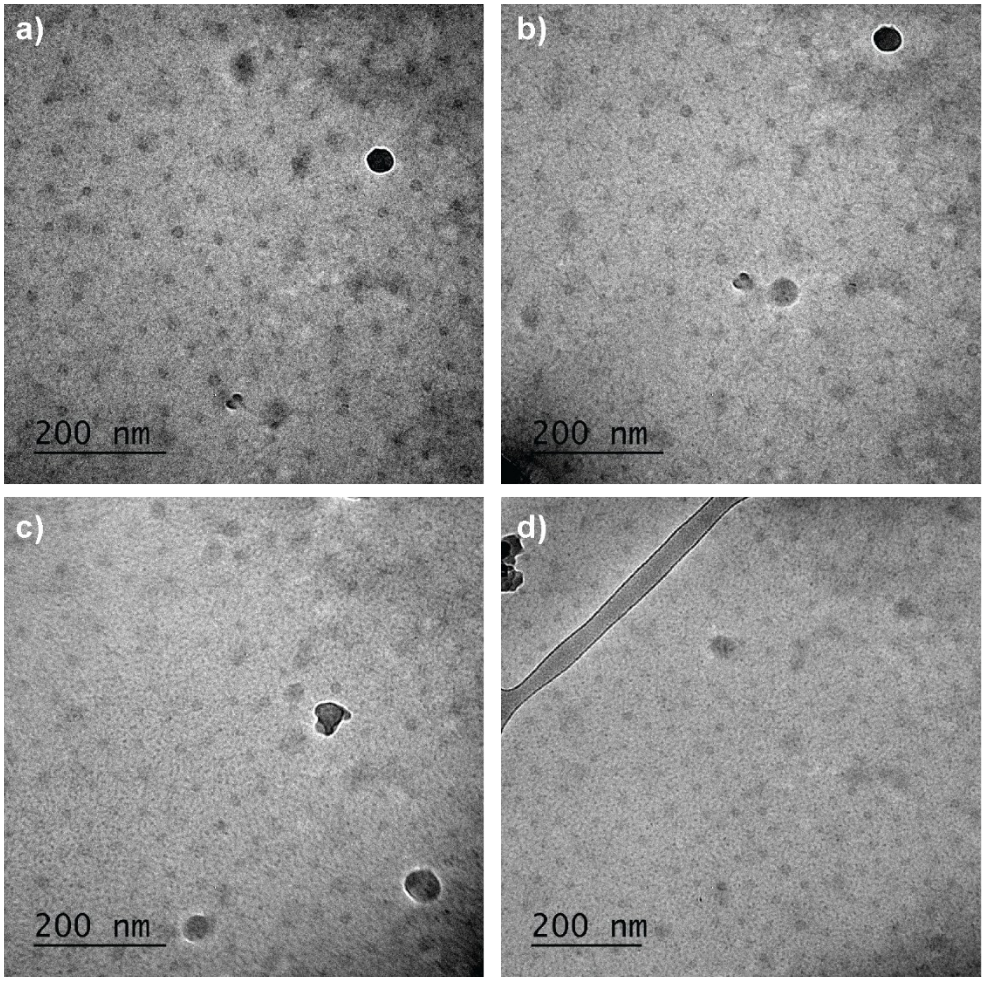

Figure S12. Cryo-TEM images of **ABC** TBC aqueous solution (1 mg/mL).

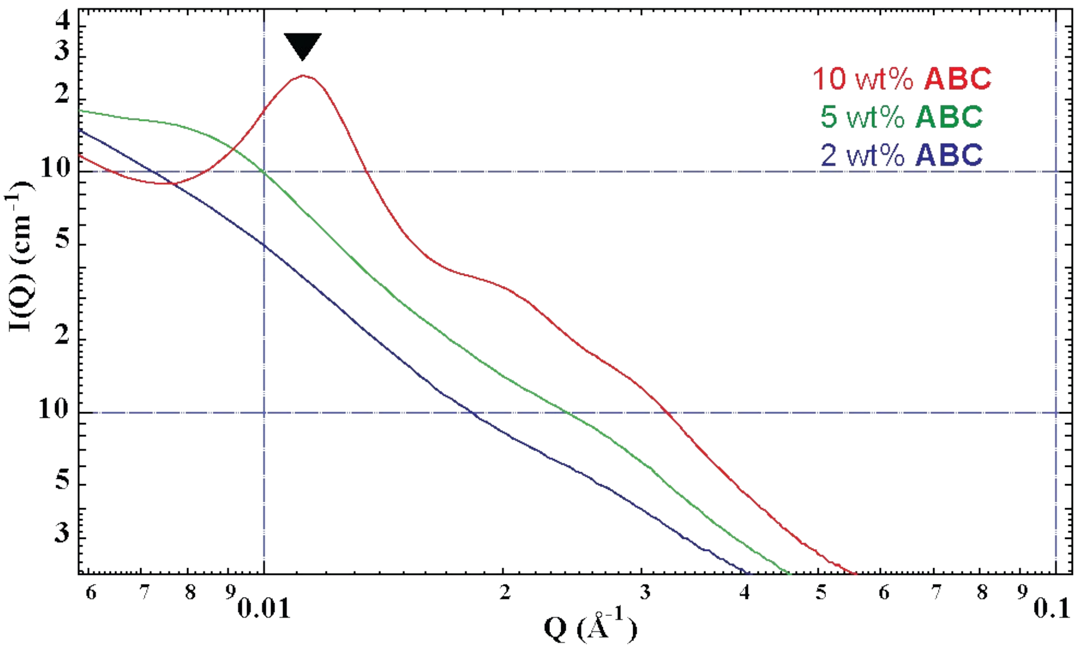

Figure S13. SAXS measurements of **ABC** TBC aqueous solutions at different concentrations (2-10 wt%) at room temperature.

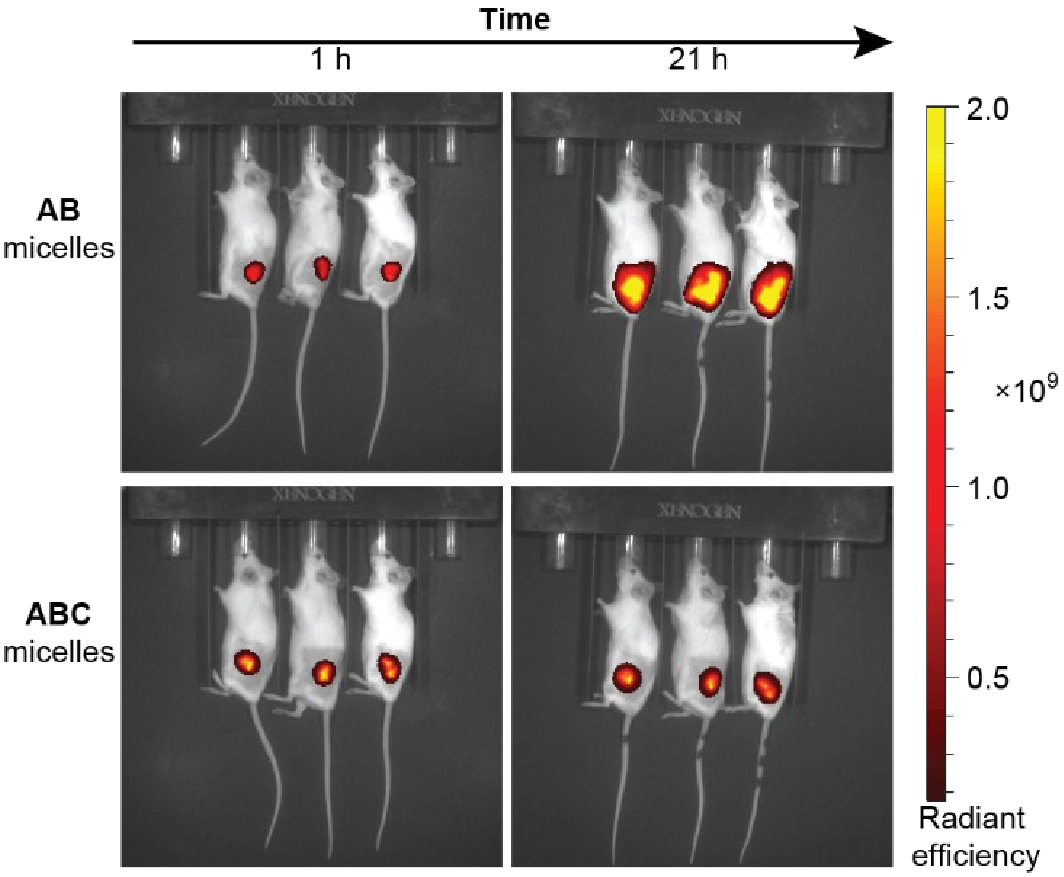

Figure S14. Fluorescence imaging (IVIS) of mice injected with **AB-Cy7.5** and **ABC-Cy7.5** (10 wt%) at 1 h and at 24 h after injection. Ex/Emm = 745/800 nm.

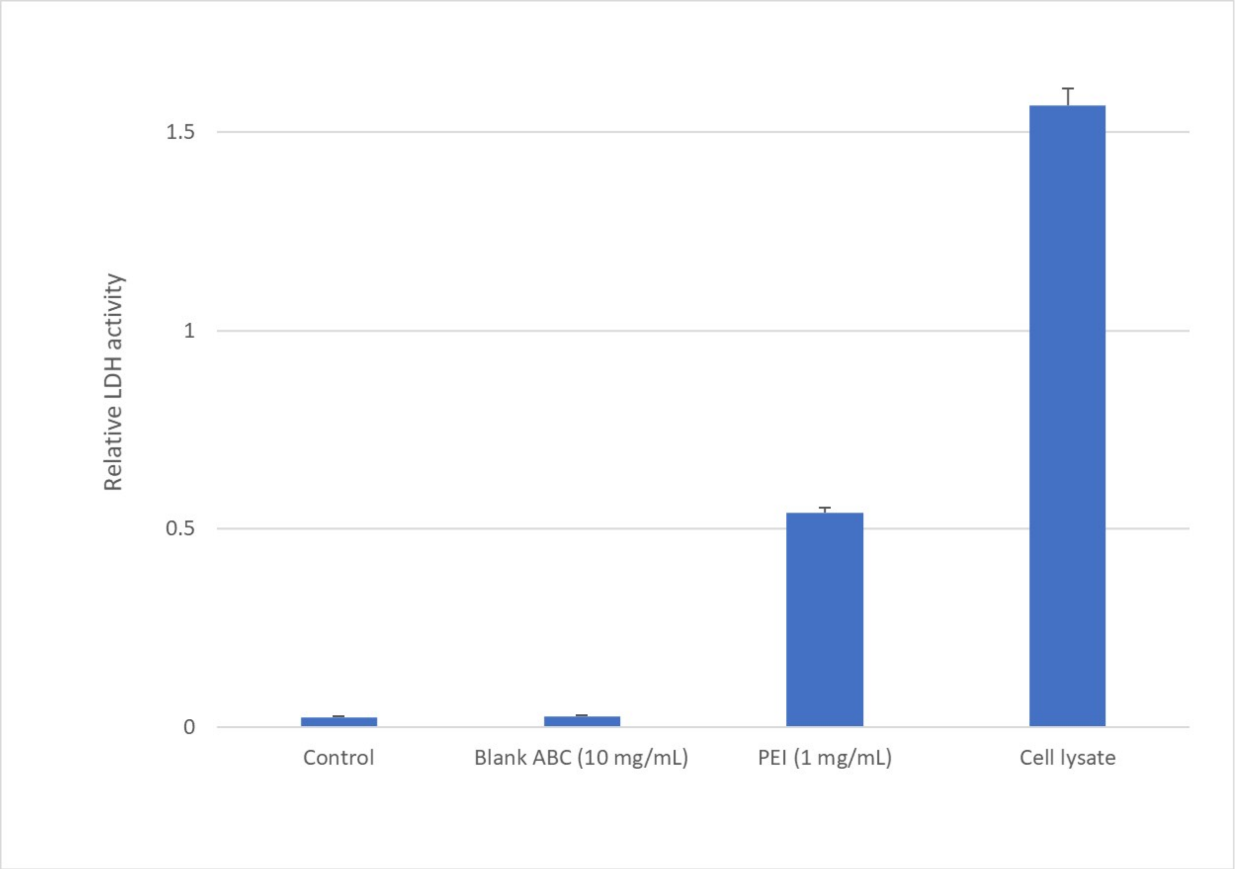

Figure S15. Assay for free lactate dehydrogenase (LDH) of human umbilical vein endothelial cells (HUVEC) incubated with **ABC** bottlebrush aqueous solution.

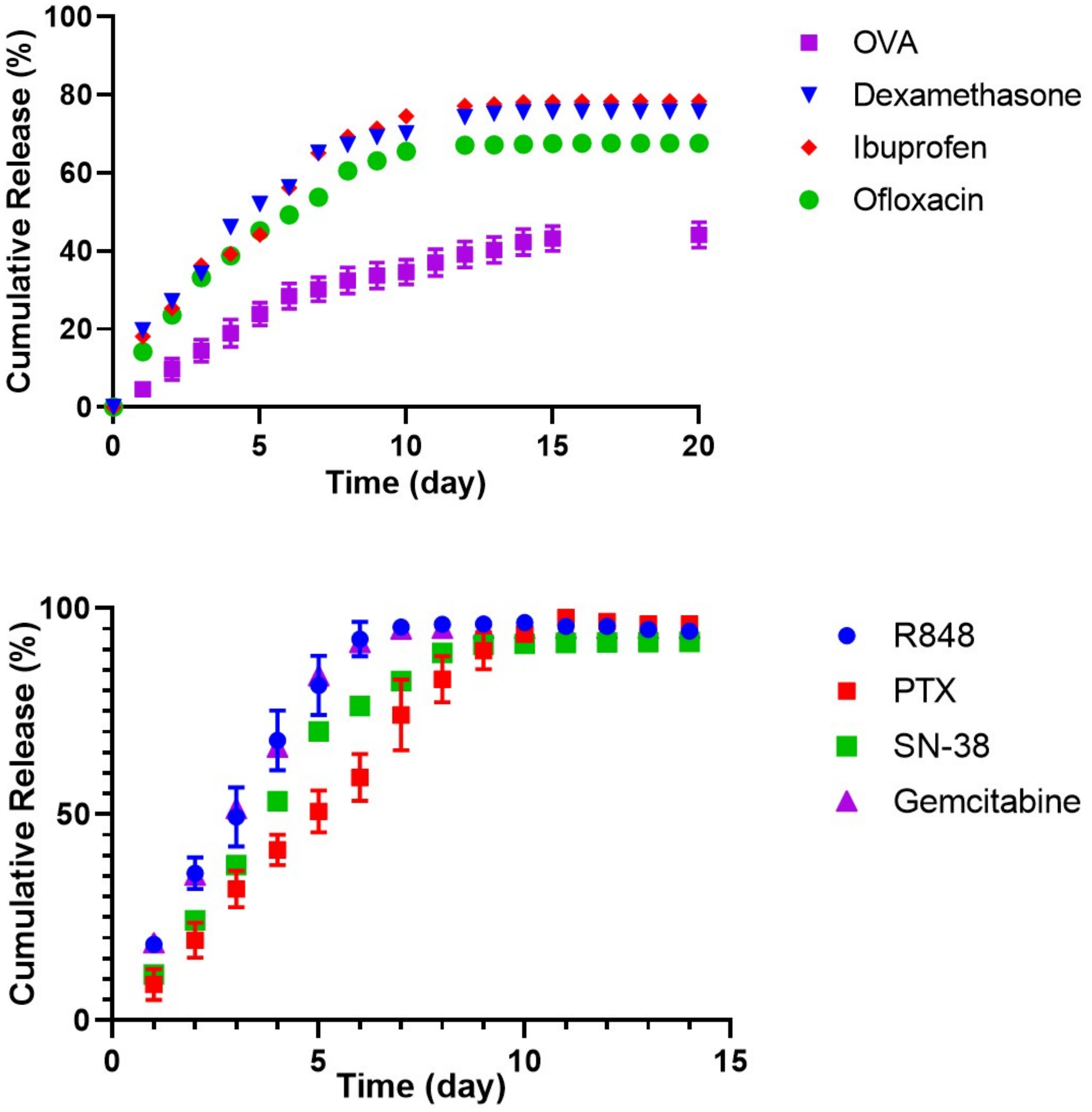

Figure S16. Sustained release from **ABC** hydrogel at 37 °C in PBS: drugs commonly used after ophthalmic surgery and a model protein, ovalbumin (Top); common chemotherapeutics and an immunomodulator (R848) (Bottom).

a)

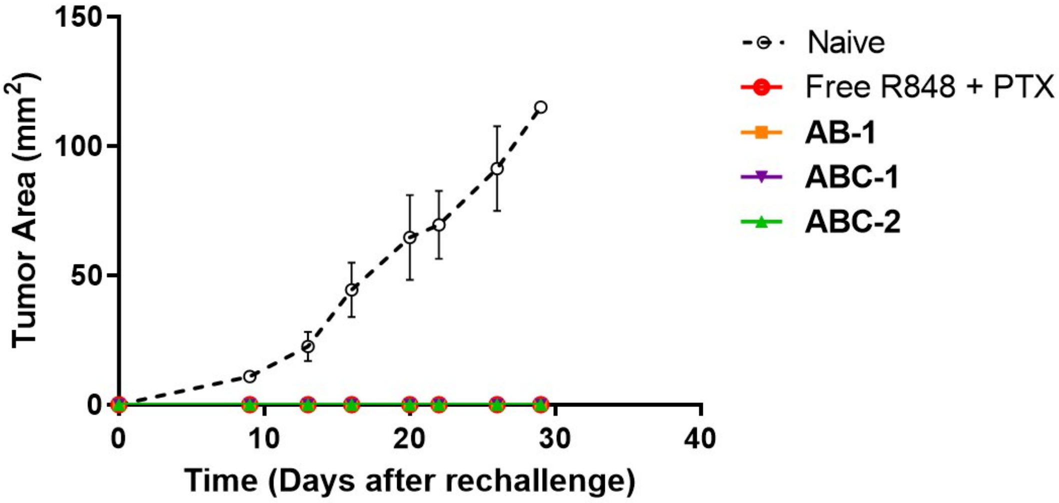

b)

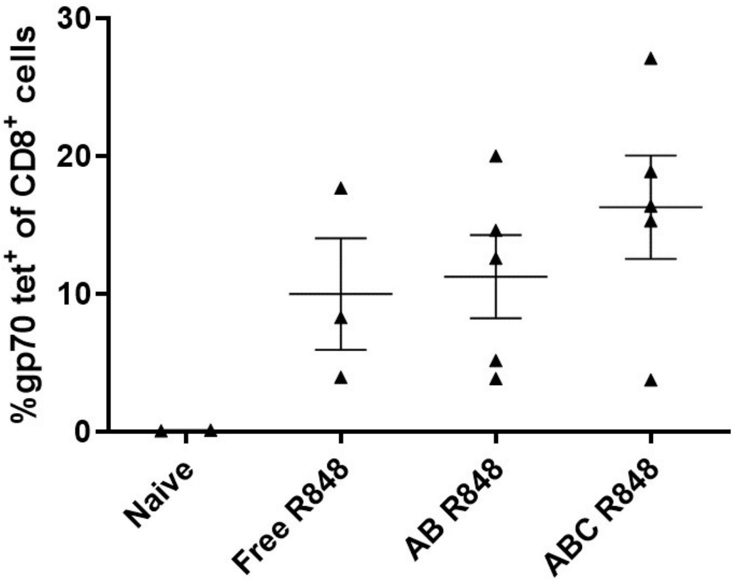

Figure S17. a) Long-time surviving mice in all R848+PTX treatment groups gained immunity against CT26. Long-term surviving mice (n = 5) from each treatment group were rechallenged with CT26 10^5 tumor cells on the opposite hind flank. As a control, naïve mice were implanted with the same number of cells and experienced rapid tumor outgrowth. b) Mice (n = 5) treated intratumorally with R848 developed tumor antigen-specific $CD8^+$ T cells, supporting the proposed mechanism of tumor rejection. Error bars represent SEM.

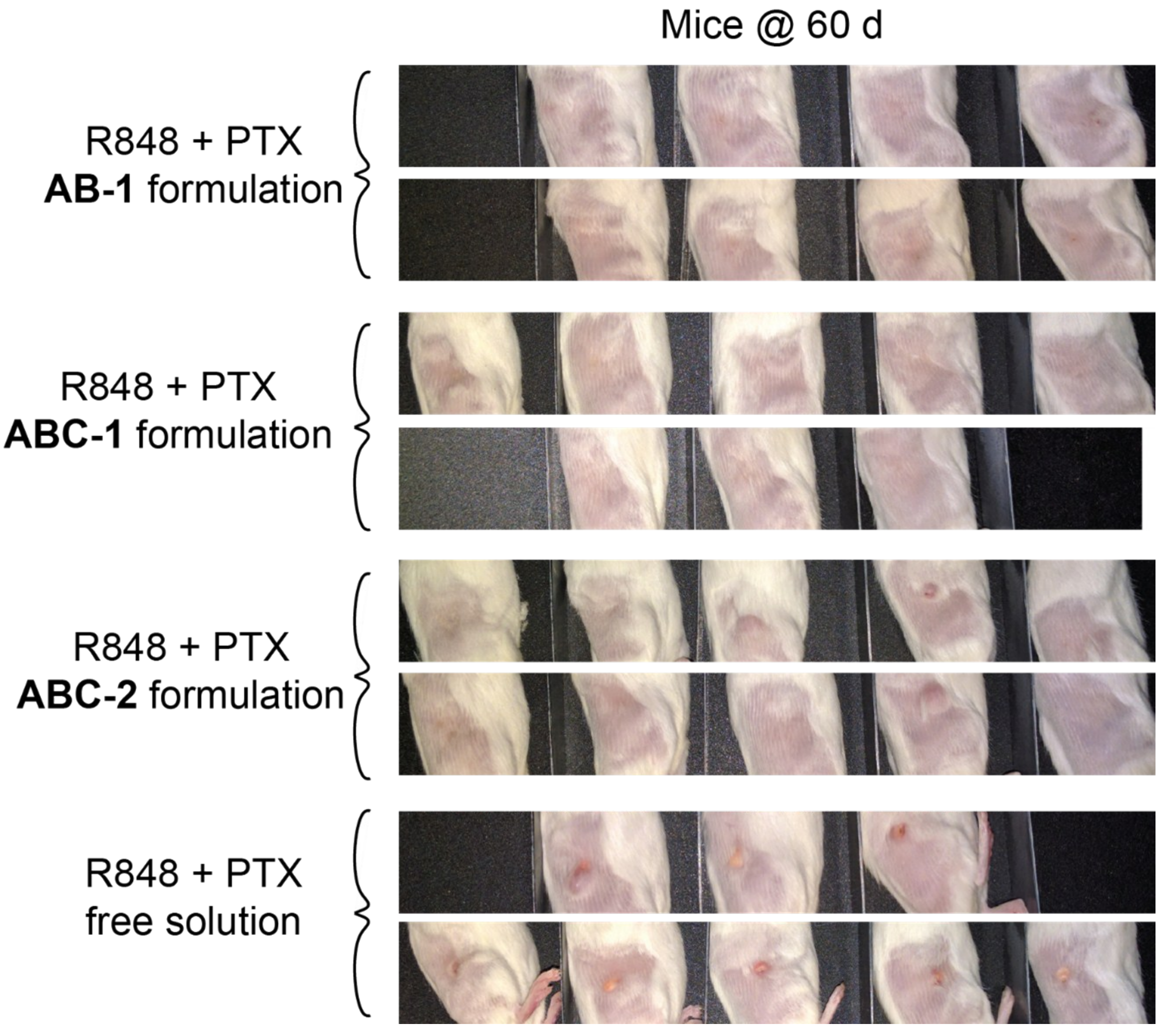

Figure S18. Mice in free R848 and PTX combination treatment group developed persistent nodules at injection sites. The nodules displayed signs of a chronic local inflammation with extensive scar tissue formation visible under healed dermis.

a)

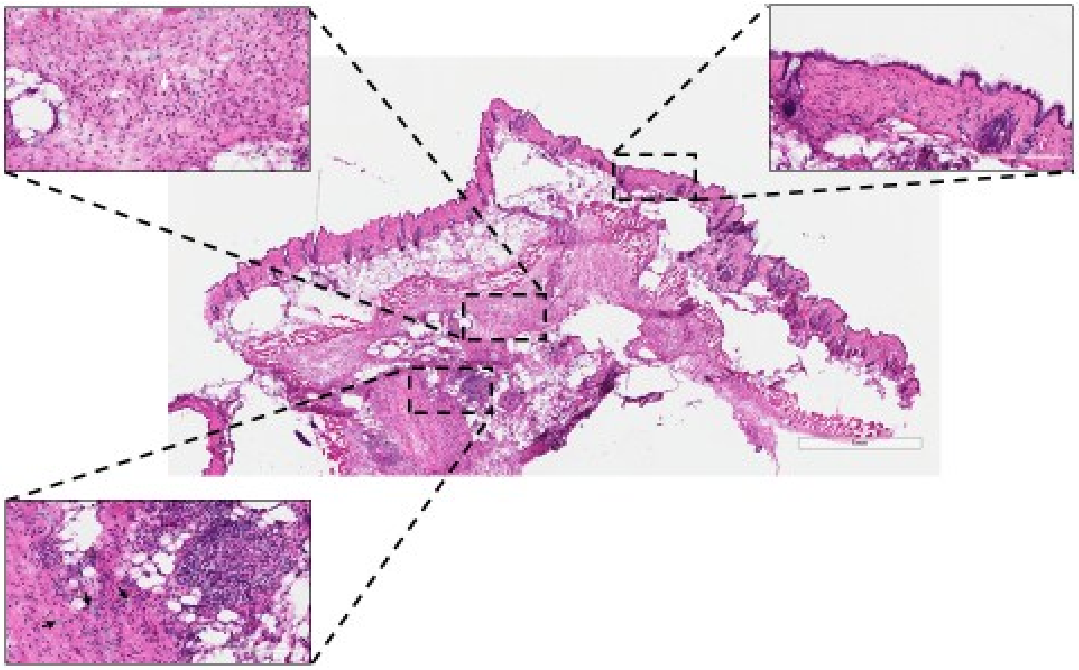

b)

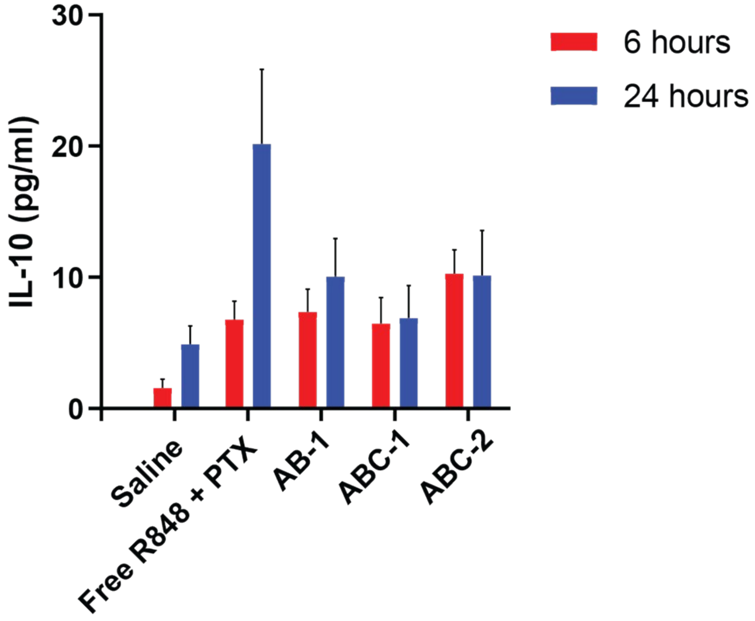

Figure S19. a) A representative tissue sample from the site of the scar formation in mice treated with free resiquimod (R848) and paclitaxel (PTX) combination. The tissue samples were collected for analysis at 60 d and show archetypal signs of chronic inflammation: i) collagen buildup, scar tissue formation, ii) increased monocyte and granulocyte infiltration, and iii) extensive presence of lymphocytes. b) Higher levels of systemic anti-inflammatory cytokine Il-10 were observed following intratumoral injection of free R848 + PTX combination.

4. Supplemental characterization data

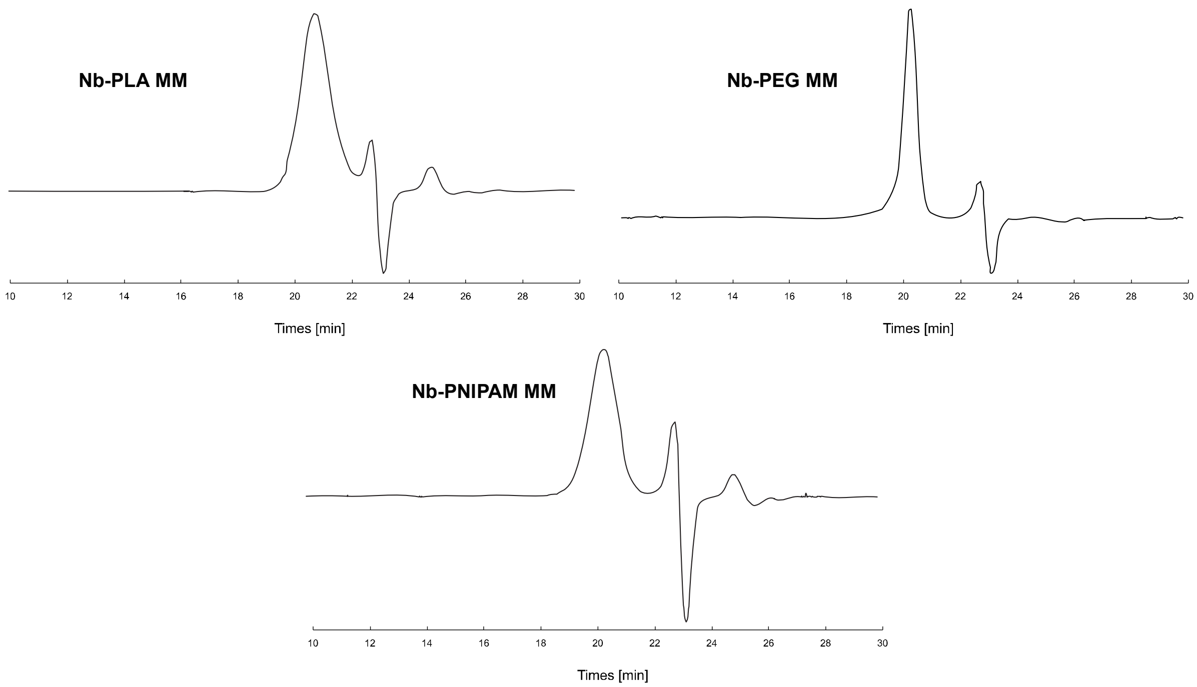

SEC analysis of macromonomers.

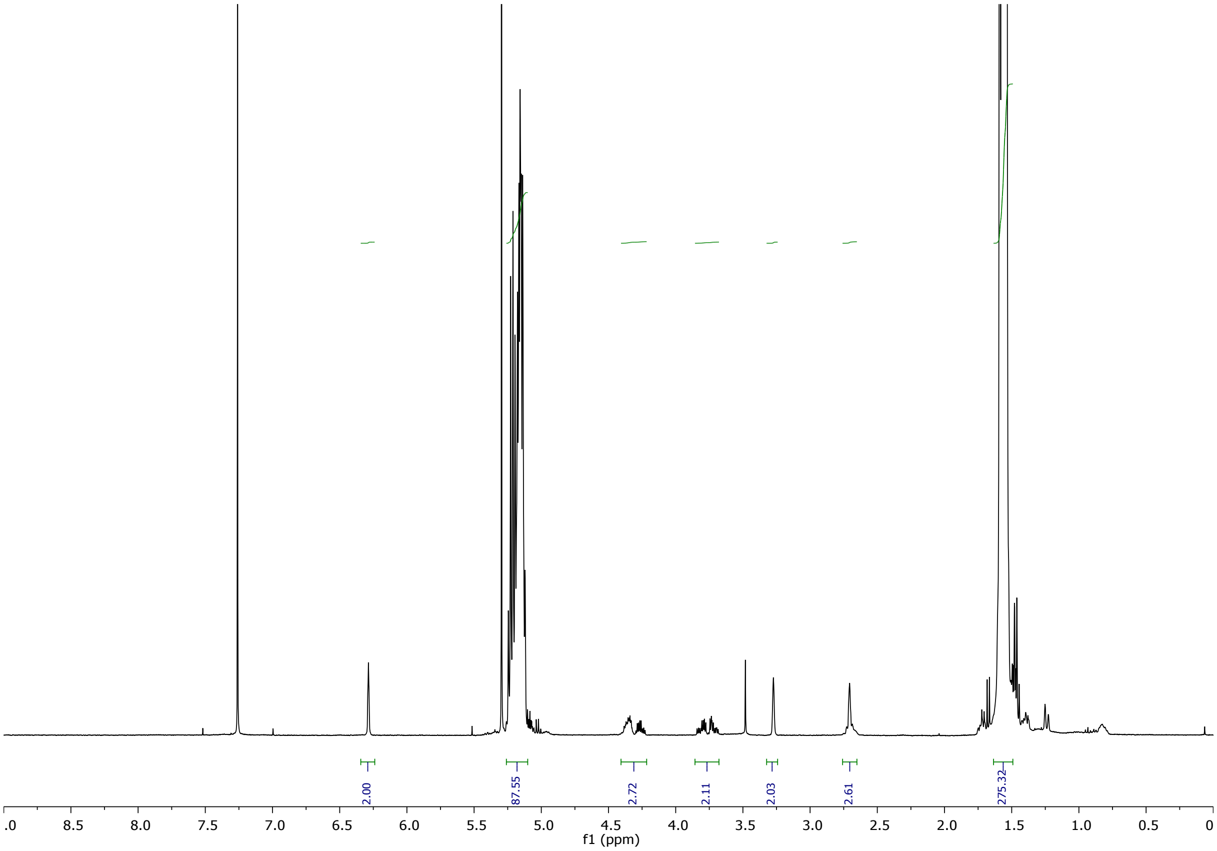

^1H NMR of **Nb-PLA** macromonomer in $CDCl_3$.

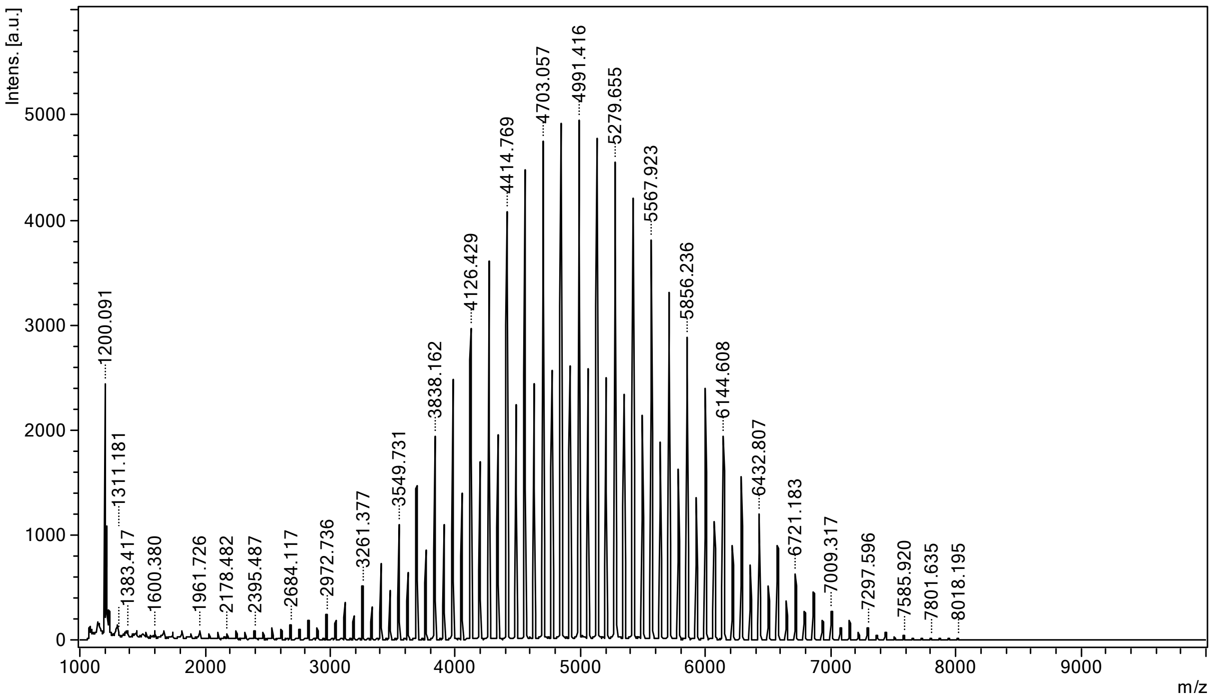

MALDI-MS of **Nb-PLA** macromonomer using dithranol matrix.

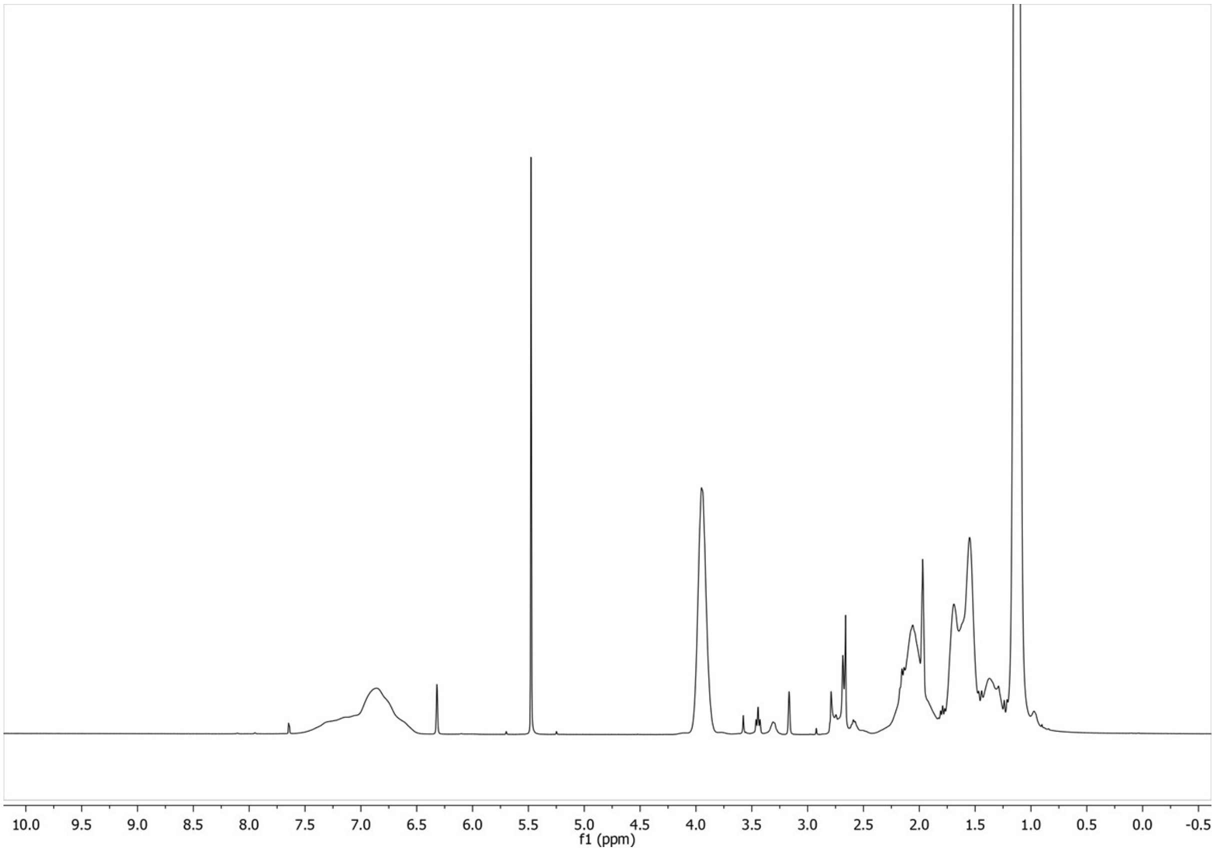

^{1}H NMR of **Nb-PNIPAM** macromonomer in CD_3CN with 5% D_2O.

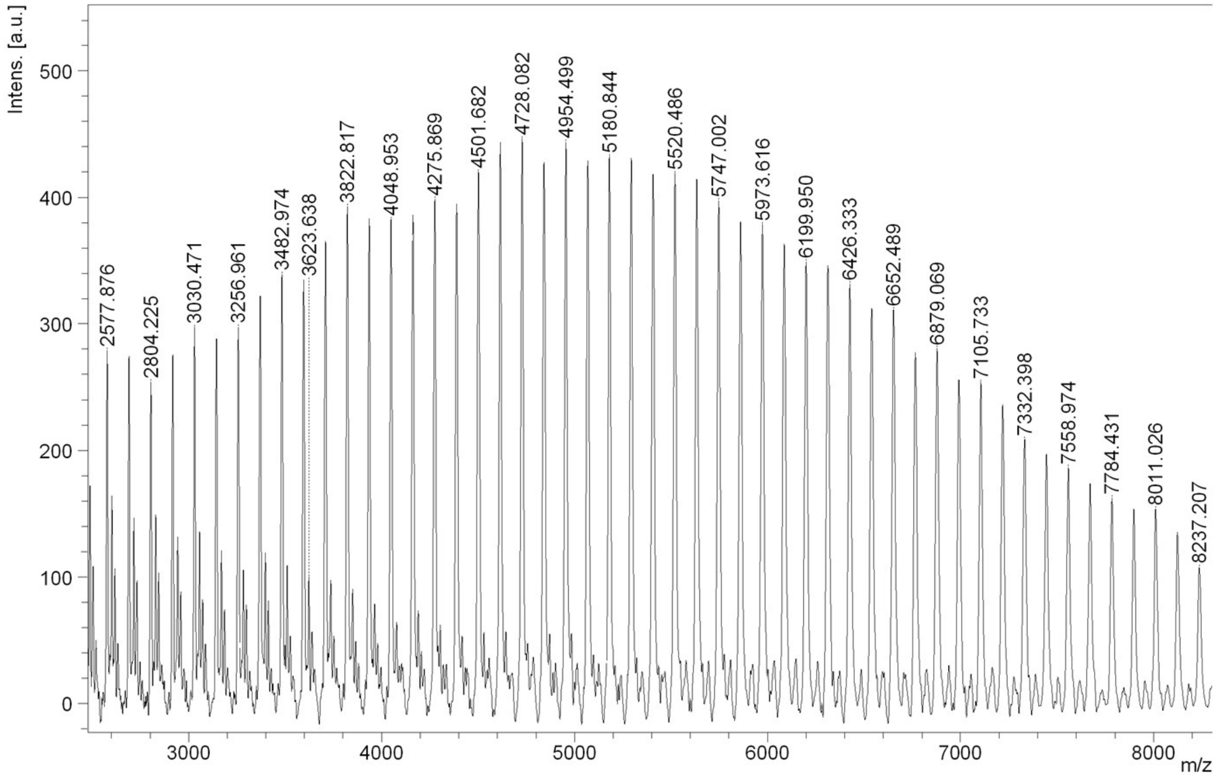

MALDI-MS of **Nb-PNIPAM** macromonomer using sinapinic acid matrix.

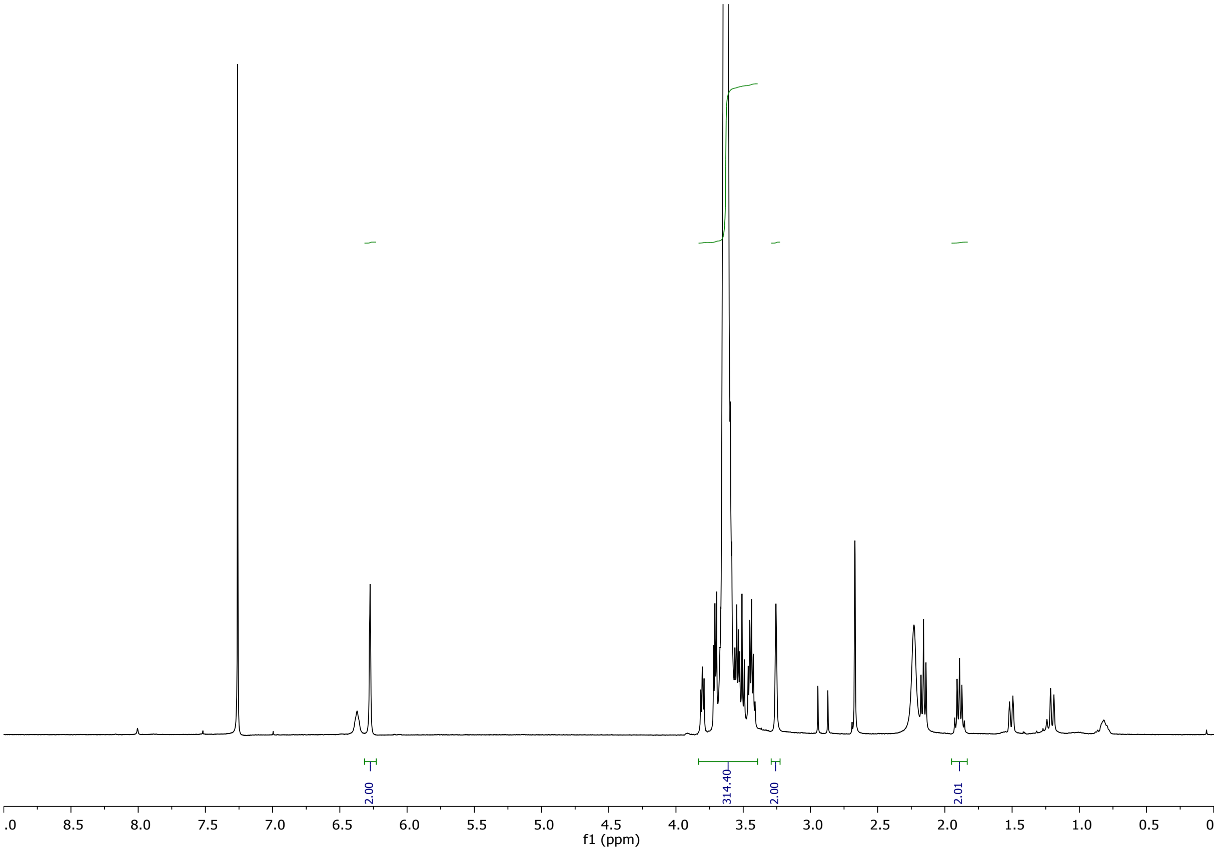

^{1}H NMR of **Nb-PEG** macromonomer in $CDCl_3$.

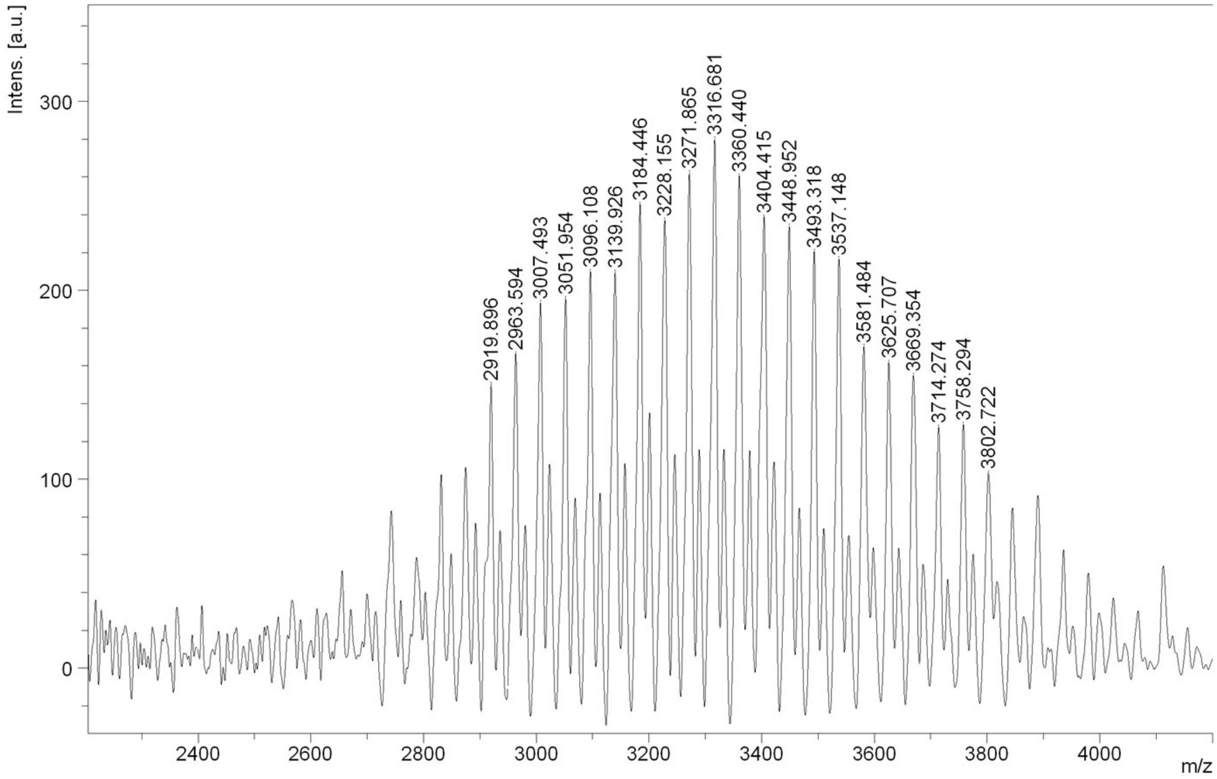

MALDI-MS of **Nb-PEG** macromonomer using sinapinic acid matrix.

5. References

(1) Love, J. A.; Morgan, J. P.; Trnka, T. M.; Grubbs, R. H., A Practical and Highly Active Ruthenium-Based Catalyst that Effects the Cross Metathesis of Acrylonitrile. *Angew. Chem., Int. Ed.* **2002,** *41*, 4035.

(2) Liu, J.; Burts, A. O.; Li, Y.; Zhukhovitskiy, A. V.; Ottaviani, M. F.; Turro, N. J.; Johnson, J. A., "Brush-First" Method for the Parallel Synthesis of Photocleavable, Nitroxide-Labeled Poly(ethylene glycol) Star Polymers. *J. Am. Chem. Soc.* **2012,** *134*, 16337.

(3) Sveinbjörnsson, B. R.; Weitekamp, R. A.; Miyake, G. M.; Xia, Y.; Atwater, H. A.; Grubbs, R. H., Rapid self-assembly of brush block copolymers to photonic crystals. *Proc. Natl. Acad. Sci. U. S. A.* **2012,** *109*, 14332.

(4) Golder, M. R.; Liu, J.; Andersen, J. N.; Shipitsin, M. V.; Vohidov, F.; Nguyen, H. V. T.; Ehrlich, D. C.; Huh, S. J.; Vangamudi, B.; Economides, K. D.; Neenan, A. M.; Ackley, J. C.; Baddour, J.; Paramasivan, S.; Brady, S. W.; Held, E. J.; Reiter, L. A.; Saucier-Sawyer, J. K.; Kopesky, P. W.; Chickering, D. E.; Blume-Jensen, P.; Johnson, J. A., Reduction of liver fibrosis by rationally designed macromolecular telmisartan prodrugs. *Nat. Biomed. Eng.* **2018,** *2*, 822.
